# Four Isotope-Labeled Recombination Pathways of Ozone Formation

**DOI:** 10.3390/molecules26051289

**Published:** 2021-02-27

**Authors:** Dmitri Babikov, Elizaveta Grushnikova, Igor Gayday, Alexander Teplukhin

**Affiliations:** 1 Department of Chemistry, Wehr Chemistry Building, Marquette University, Milwaukee, WI 53201-1881, USA; elizaveta.grushnikova@mu.edu (E.G.); igor.gayday@mu.edu (I.G.); 2 Theoretical Division (T-1, MS B221), Los Alamos National Laboratory, Los Alamos, NM 87545, USA; tepl@lanl.gov

**Keywords:** ozone, isotope effect, scattering resonances, hyperspherical coordinates

## Abstract

A theoretical approach is developed for the description of all possible recombination pathways in the ozone forming reaction, without neglecting any process *a priori*, and without decoupling the individual pathways one from another. These pathways become physically distinct when a rare isotope of oxygen is introduced, such as ^18^O, which represents a sensitive probe of the ozone forming reaction. Each isotopologue of O_3_ contains two types of physically distinct entrance channels and two types of physically distinct product wells, creating four recombination pathways. Calculations are done for singly and doubly substituted isotopologues of ozone, eight rate coefficients total. Two pathways for the formation of asymmetric ozone isotopomer exhibit rather different rate coefficients, indicating large isotope effect driven by ΔZPE-difference. Rate coefficient for the formation of symmetric isotopomer of ozone (third pathway) is found to be in between of those two, while the rate of insertion pathway is smaller by two orders of magnitude. These trends are in good agreement with experiments, for both singly and doubly substituted ozone. The total formation rates for asymmetric isotopomers are found to be somewhat larger than those for symmetric isotopomers, but not as much as in the experiment. Overall, the distribution of lifetimes is found to be very similar for the metastable states in symmetric and asymmetric ozone isotopomers.

## 1. Introduction

The recombination reaction that produces the usual (isotopically unsubstituted) ozone molecule may look relatively simple, O + O_2_ → O_3_, but this is because in this case only one, the most abundant isotope of oxygen (^16^O) is involved. When a rare isotope is introduced, say ^18^O, several physically distinct reaction pathways (1)–(4) can be identified [1,2,3,4,5,6,7,8]:
*A*:      ^16^O + ^16^O^18^O →^16^O^16^O^18^O      (1)*B*:      ^18^O + ^16^O^16^O →^18^O^16^O^16^O      (2)*S*:      ^16^O + ^18^O^16^O →^16^O^18^O^16^O      (3)*I*:      ^18^O + ^16^O^16^O →^16^O^18^O^16^O      (4)

Two of these pathways, *A* and *B*, produce an asymmetric ozone molecule ^16^O^16^O^18^O, while the other two pathways, *S* and *I*, produce symmetric ozone molecules ^16^O^18^O^16^O. Three of them: *A*, *B* and *S*, correspond to the addition of an atom to a diatomic moiety (a direct end-on processes), whereas the last one, *I*, represents the insertion of a rare isotope in-between the two atoms of the reagent molecule (and thus is expected to follow a more complicated indirect reaction path, see below). If two ^18^O isotopes are involved, then four more recombination pathways need to be considered:
*A*:      ^18^O + ^18^O^16^O →^18^O^18^O^16^O      (5)*B*:      ^16^O + ^18^O^18^O →^16^O^18^O^18^O      (6)*S*:      ^18^O + ^16^O^18^O →^18^O^16^O^18^O      (7)*I*:      ^16^O + ^18^O^18^O →^18^O^16^O^18^O      (8)

with similar nomenclature, similar symmetry properties, and similar reaction mechanisms. However, as we will discuss further below, some properties of the reaction pathways (5)–(8) for doubly substituted ozone molecules appear to be just opposite to those of pathways (1)–(4) for singly substituted ozone molecules. The singly and doubly substituted molecules are said to represent two *isotopologues* of ozone. 

Different isotopologues are relatively independent, in a sense that they cannot interconvert one into another, although some of the reagents they use are the same, namely, the ^16^O and ^18^O atoms, and the ^16^O^18^O diatomic, see Equations (1)–(8). However, within each isotopologue the symmetric and asymmetric ozone molecules are closely related, since they can interconvert one into another, at least in principle, following an isomerization path through the potential energy surface, for example:

(9) O16O16O18⇋O16O18O16

or:

(10) O18O16O18⇋O16O18O18

**Figure 1:**
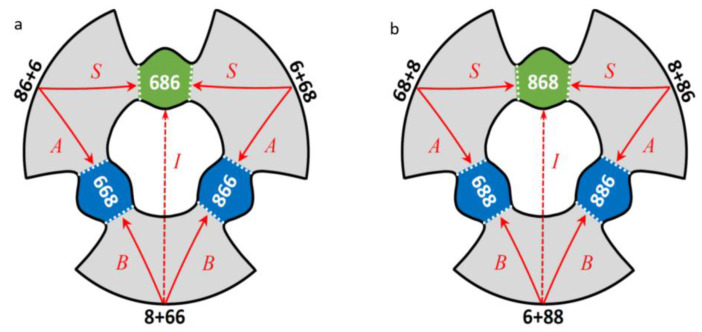
Schematic of the global PES of ozone that possesses a three-fold symmetry with respect to the entrance channels and the product wells, but this symmetry is lowered when rare isotopes are introduced. The channels and the wells are labeled for singly substituted (**a**) and doubly substituted (**b**) isotopologues, respectively. Reagents are indicated by black numbers, the product ozone molecules by white numbers, dotted lines are drawn through transition state regions seen as “bottlenecks” in this figure. Four distinguishable reaction pathways are also indicated in each case, by red letters and arrows. The insertion pathway is shown schematically by dashed arrow.

Therefore, symmetric and asymmetric ozone molecules within the same isotopologue are called *isotopomers*. In Figure 1a,b, a schematic of the potential energy surface is given for each isotopologue, with two isotopomers in each case, and four reaction pathways indicated.

From this figure one can see that overall, on the global potential energy surface of a triatomic ozone molecule, there are three entrance channels, connected through six transition states to three potential energy wells. However, since two isotopes in each case are indistinguishable (two ^16^O in the singly substituted isotopologue, or two ^18^O in the doubly substituted isotopologue) there are only *two* distinguishable sets of reagents, and *two* distinguishable products, leading to *four* distinguishable reaction pathways in each case: *A*, *B* and *S* and *I*, as introduced above. For the purpose of brevity, here and below, the notations “6” and “8” will be used to designate isotopes ^16^O and ^18^O respectively. Note that 668 and 866 indicate physically indistinguishable asymmetric ozone molecules formed simultaneously and counted in together as one product. The same is true for 688 and 886 in the case of double substitution. 

Experimental studies started by the Mauersberger group [1,2,3,4,5] and continued by Janssen and coworkers [6,7,8], gave us unique information about the magnitudes of recombination rate coefficients for these four pathways: *κ*_Α_, *κ*_B_, *κ*_S_ and *κ*_I_ in both singly and doubly substituted cases. Their results, summarized in Table 1 here, indicate a broad variation of recombination rate coefficients through isotopomers and isotopologues of ozone (the factor of 2 is introduced for *κ*_Α_ and *κ*_S_ to account for the usual symmetry effect associated with the number of states in the homonuclear and heteronuclear diatomic reagents). In the first row we collected the values of 2*κ*_S_ that can be used as a convenient reference for each column (Indeed, note that the values of 2*κ*_S_ are very close to each other and to the experimental value for *κ*_666_ = 60 × 10^−35^ cm^6^/s for the usual unsubstituted ozone) [9]. The values of rate coefficients 2*κ*_Α_ and *κ*_B_ collected in the second and third rows deviate significantly from 2*κ*_S_, and, importantly, these deviations occur in the opposite directions in the cases of single and double isotopic substitutions (compare even columns with odd columns of Table 1). Finally, the values of *κ*_I_ for the insertion pathway, listed in the fourth row, are much smaller than the other three, by up to two orders of magnitude. 

These experimentally measured rate coefficients suggest the presence of three definite isotope related phenomena, namely (see Table 1):

(1) Quantum ΔZPE-effect, responsible for large difference, about 60%, between 2*κ*_Α_ and *κ*_B_ of the pathways *A* and *B* that are just slightly endo/exothermic (one with respect to another). This phenomenon will be named *ζ*-effect.
(2) Symmetry-driven η-effect [1,10,11,12], that favors formation of asymmetric molecules, ^16^O^16^O^18^O and ^16^O^18^O^18^O, compared to the symmetric ones, ^16^O^18^O^16^O and ^18^O^16^O^18^O, by about 16%;
(3) Very large difference, by a factor of ~5, between the insertion rate coefficients *κ*_I_ in the singly and doubly substituted cases [1]. Here we will call it ξ-effect.

The first two effects received a lot of attention from the theory community [13,14,15,16,17,18,19,20,21,22,23,24]. First, the large ΔZPE-effect was shown to originate from quantum mechanics [14,15,16] and thus is fundamentally important for our understanding of the chemical reaction rate laws in general. Nowadays this phenomenon is understood relatively well [17,20,21,22] and will naturally emerge as a part of the theory developed below. The symmetry-driven η-effect, practically important for atmospheric chemistry [6,12], remains mysterious, despite of significant efforts dedicated to finding its origin [18,19,20,21,22,23,24]. One of the goals of this work is to develop theoretical grounds for the description of this effect (although at present time we cannot say that we entirely understand it). In sharp contrast with the first two phenomena, the third and the largest isotope effect, related to the insertion pathway in singly and doubly substituted isotopologues, has never been discussed in the literature. 

Indeed, the rate coefficient *κ*_I_ of insertion has never been computed theoretically by anyone. One reason for this is that in the experiment the values of *κ*_I_ are smaller than those of *κ*_Α_*,* *κ*_B_ and *κ*_S_, roughly, by two orders of magnitude [1], and therefore for theorists it is very tempting to neglect *κ*_I_. But then the relevant isotope effect is also lost. In this work we develop a theory that includes all pathways of ozone formation, without neglecting anything *a priori*. 

Another reason for the absence of any theoretical work on the insertion pathway is that this process is not a simple direct reaction. In the Figure 1 above, for simplicity, this process is shown by a straight arrow but, in reality, the reaction path cannot cross the center of this figure (at any relevant temperature) since the PES is extremely repulsive in that part of the configuration space. The insertion path must be indirect, akin to the roaming mechanism [25], when the reagents enter through one channel, form an excited complex in the adjacent well, then roam through the other channel, isomerizing into the other well, where the second complex is formed and finally stabilized by bath gas collisions. For example, in the case of singly substituted isotopologue this process can be represented as:

(11) O18+ O16O16 → O18⋯O16O16 →dec  O18O16⋯O16 →roam O16⋯ O18O16  →stab O16O18O16

In the doubly substituted case:

(12) O16+ O18O18 →form O16⋯O18O18 →dec  O16O18⋯O18 →roam O18⋯ O16O18  →stab O18O16O18

How to describe these processes theoretically? Classical trajectories are used for the description of roaming [25], but the classical approach is efficient and accurate only if roaming is the dominant mechanism of the reaction, or the only one possible. In the case of ozone, the insertion by roaming is a minor pathway, with a small probability. Prediction of rare events using classical trajectories is problematic on its own, and therefore is not expected to be helpful in the case of ozone. The quantum dynamics treatment, such as the wave packet method [26], is usually employed for the description of direct reactions, that proceed through the transition state relatively fast, without formation of a long-lived complex. The propagation of wave packets through the extended parts of the potential energy landscape, in particular over the deep wells where the long-lived complexes (scattering resonances) are formed, is numerically challenging [26]. Therefore, the quantum dynamical description of roaming is not routinely done [27]. It appears that all previously developed theoretical models of ozone formation either used an approximate treatment for scattering resonances or did not include the lifetimes at all, appealing to the low-pressure regime [28,29,30,31].

In this work, for theoretical description of the insertion process, we employed a general time-independent quantum mechanical approach, in which the metastable ozone states above dissociation threshold are described by scattering resonances. Complex absorbing potential is introduced in the asymptotic part of the PES, and the widths of individual scattering resonances $\Gamma_{i}$ are obtained from the complex parts of the total energy, that are used to compute rate coefficients of decay (spontaneous, first order) of the individual scattering resonances as:

(13) $$k_{i}^{\mathrm{dec}}\;=\;\frac{\Gamma_{i}}{\hbar }$$

Importantly, within this approach, we can setup the complex potential in the individual channels on the PES, in order to split the resonance width and the corresponding decay rate coefficient onto two channel-specific contributions:

(14) $$\Gamma_{i}^{\mathrm{tot}}=\Gamma_{i}^{\mathrm{ch}1}+\Gamma_{i}^{\mathrm{ch}2}$$

(15) $$k_{i}^{\mathrm{dec}}=\;k_{i}^{\mathrm{dec},\mathrm{ch}1}+k_{i}^{\mathrm{dec},\mathrm{ch}2}$$

Here $k_{i}^{\mathrm{dec},\mathrm{ch}1}=\Gamma_{i}^{\mathrm{ch}1}/\hbar$, whereas $k_{i}^{\mathrm{dec},\mathrm{ch}2}=\Gamma_{i}^{\mathrm{ch}2}/\hbar$. For example, in the singly substituted case $k_{i}^{\mathrm{dec},\mathrm{ch}1}$ corresponds to the decay of metastable ozone onto ^18^O + ^16^O^16^O through one channel at the bottom of Figure 1a, while $k_{i}^{\mathrm{dec},\mathrm{ch}2}$ corresponds to the decay of a metastable ozone onto ^16^O + ^16^O^18^O through two indistinguishable channels at the top of Figure 1a. Similar, in the doubly substituted case $k_{i}^{\mathrm{dec},\mathrm{ch}1}$ corresponds to the decay of ozone onto ^16^O + ^18^O^18^O through one channel at the bottom of Figure 1b, while $k_{i}^{\mathrm{dec},\mathrm{ch}2}$ corresponds to the decay of a resonance onto ^18^O + ^18^O^16^O through two indistinguishable channels at the top of Figure 1b. Using the equilibrium constant (computed statistically), the rate coefficients of *decay* can be converted into the rate coefficients for *formation* of the metastable ozone states. Note that this part of the method is based on rigorous quantum mechanical calculations.

The second feature of our theory is the splitting of the rate coefficient for *stabilization* of scattering resonances (second order, by collisions with bath gas) onto two components - those that produce symmetric and asymmetric ozone molecules. This is done semi-empirically, by assuming that stabilization rate coefficient for a scattering resonance is proportional to the probability amplitude over certain area of the PES. Namely, integrating the moduli squared of wavefunctions of the individual scattering resonances over the parts of the PES associated with symmetric and asymmetric ozone molecules (see Figure 1), we can determine the product-specific probabilities $p_{i}^{\mathrm{sym}}$ and $p_{i}^{\mathrm{asym}}$, and the total probability [21,22,23,24]:

(16) $$p_{i}^{\mathrm{tot}}=\;p_{i}^{\mathrm{sym}}+p_{i}^{\mathrm{asym}}$$

The two product-specific stabilization rate coefficients, are then computed as:

(17) $$k_{i}^{\mathrm{stab},\;\mathrm{sym}}=\;k^{\mathrm{stab}}p_{i}^{\mathrm{sym}}$$

(18) $$k_{i}^{\mathrm{stab},\;\mathrm{asym}}=\;k^{\mathrm{stab}}p_{i}^{\mathrm{asym}}$$

The values of probabilities $p_{i}^{\mathrm{sym}}$ and $p_{i}^{\mathrm{asym}}$ are rigorously determined for individual scattering resonances, by integrating their wave functions. In the singly substituted case $p_{i}^{\mathrm{sym}}$ and $p_{i}^{\mathrm{asym}}$ are associated with ^16^O^18^O^16^O and ^16^O^16^O^18^O, while in the doubly substituted case they correspond to ^18^O^16^O^18^O and ^16^O^18^O^18^O, respectively. Their sum defines the total stabilization rate coefficient, naturally, as $k_{i}^{\mathrm{stab}}\;=k^{\mathrm{stab}}p_{i}^{\mathrm{tot}}$. One single value of $k^{\mathrm{stab}}=\;v\sigma_{\mathrm{stab}\;}$, where $v=\sqrt{8kT/\left(\pi \mu \right)}$, *μ* is reduced mass and σ_stab_ = 154.03 $a_{0}^{2}$ is used for all resonances, as was recommended earlier in the mixed quantum/classical study of collisional energy transfer [32].

Combination of the two *channel-specific* rate coefficients for decay of resonances, with two *product-specific* stabilization rate coefficients, permits to express four *pathway-specific* recombination rate coefficients *κ*_Α_*,* *κ*_B_, *κ*_S_ and *κ*_I_ through $\Gamma_{i}^{\mathrm{ch}1}$, $\Gamma_{i}^{\mathrm{ch}2}$, $p_{i}^{\mathrm{sym}}$ and $p_{i}^{\mathrm{asym}}$ (for each resonance). In addition, we will obtain, analyze and employ the expressions for recombination rate coefficients *κ*_sym_ and *κ*_asym_ for production of symmetric and asymmetric ozone molecules. 

## 2. Theory

### 2.1. The Four Pathways of the Recombination Reaction

According to the Lindemann mechanism [33], the rate *R* of a recombination process is a product of the concentration of metastable species $\left[O_{3}^{*}\right]$ represented here by scattering resonances, the concentration of bath gas particles [M], and the stabilization rate coefficient *k*^stab^, see Equation (19). If we assume that different resonances are populated and stabilized independently, the total rate is given by the sum over scattering resonances (labelled by index *i*):

(19) $$R\;=\underset{i}{\sum }k_{i}^{\mathrm{stab}}\left[M\right]{\left[O_{3}^{*}\right]}_{i}$$

Using Equation (16) this formula splits onto two contributions that correspond to the rates of production of symmetric and asymmetric ozone molecules, as follows:

(20) $$R\;=\;\underset{i}{\sum }k^{\mathrm{stab}}\left(p_{i}^{\mathrm{sym}}+\;p_{i}^{\mathrm{asym}}\right)\left[M\right]\;{\left[O_{3}^{*}\right]}_{i}\;=\;k^{\mathrm{stab}}\left[M\right]\underset{i}{\sum }{\left[O_{3}^{*}\right]}_{i}p_{i}^{\mathrm{sym}}\;+\;k^{\mathrm{stab}}\left[M\right]\underset{i}{\sum }{\left[O_{3}^{*}\right]}_{i}p_{i}^{\mathrm{asym}}$$

If we define the corresponding rates as:

(21) $$R_{\mathrm{sym}\;}=k^{\mathrm{stab}}\left[M\right]\underset{i}{\sum }{\left[O_{3}^{*}\right]}_{i}p_{i}^{\mathrm{sym}}$$

(22) $$R_{\mathrm{asym}\;}\;=\;k^{\mathrm{stab}}\left[M\right]\underset{i}{\sum }{\left[O_{3}^{*}\right]}_{i}p_{i}^{\mathrm{asym}}$$

then the total rate is simply the sum of two:

(23) $$R_{\mathrm{tot}}=R_{\mathrm{sym}}+R_{\mathrm{asym}}$$

Note that summation in Equations (21) and (22) goes through all resonances, through exactly the same set of states in both Equations (21) and (22). What differentiates *R*_sym_ from *R*_asym_ are the values of stabilization probabilities defined for each state *i*: $p_{i}^{\mathrm{sym}}$ in Equation (21) versus $p_{i}^{\mathrm{asym}}$ in Equation (22). Some states may contribute exclusively to *R*_sym_, others exclusively to *R*_asym_, while some resonances with delocalized wavefunctions may contribute to both *R*_sym_ and *R*_asym_ with certain probabilities. Our description is general enough to incorporate all these cases.

Here we will consider the case of single isotopic substitution (the case of double substitution is similar and is presented in the Supplementary Materials). Since two physically distinct reaction channels are present for each isotopologue, the concentration of metastable ozone species $\left[O_{3}^{*}\right]$ is influenced by six processes indicated in the following diagram (for the singly substituted case): 
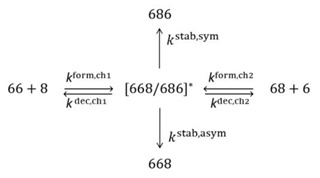
(24)

This includes the processes of formation and decay through each channel, and the processes of stabilization by bath gas collisions into the stable ozone molecules. These processes can be accounted for as follows:

(25) $$\frac{d{\left[O_{3}^{*}\right]}_{i}}{dt}=k_{i}^{\mathrm{form},\mathrm{ch}1}\left[66\right]\left[8\right]+k_{i}^{\mathrm{form},\mathrm{ch}2}\left[68\right]\left[6\right]\;-\;k_{i}^{\mathrm{dec},\mathrm{ch}1}{\left[O_{3}^{*}\right]}_{i}\;-\;k_{i}^{\mathrm{dec},\mathrm{ch}2}[O_{3}^{*}{]}_{i}-k_{i}^{\mathrm{stab},\mathrm{sym}}\left[M\right][O_{3}^{*}{]}_{i}-k_{i}^{\mathrm{stab},\mathrm{asym}}\left[M\right]{\left[O_{3}^{*}\right]}_{i}$$

Employing the steady state approximation, and, using Equations (15), (17) and (18) for the total rates of decay and stabilization, we obtain:

(26) $${\left[O_{3}^{*}\right]}_{i}=\frac{k_{i}^{\mathrm{form},\mathrm{ch}1}\left[66\right]\left[8\right]+k_{i}^{\mathrm{form},\mathrm{ch}2}\left[68\right]\left[6\right]}{k_{i}^{\mathrm{dec}}+k_{i}^{\mathrm{stab}}\left[M\right]\;}$$

It is convenient to introduce, for each scattering resonance, the equilibrium constants for two entrance channels, as follows:

(27) $$K_{i}^{\mathrm{eq},\mathrm{ch}1}=\frac{k_{i}^{\mathrm{form},\mathrm{ch}1}}{k_{i}^{\mathrm{dec},\mathrm{ch}1}\;}$$

(28) $$K_{i}^{\mathrm{eq},\mathrm{ch}2}=\frac{k_{i}^{\mathrm{form},\mathrm{ch}2}}{k_{i}^{\mathrm{dec},\mathrm{ch}2}\;}$$

This permits to express concentration through the rate of decay only (which is a fundamental property of a resonance, related to its width):

(29) $${\left[O_{3}^{*}\right]}_{i}=\frac{k_{i}^{\mathrm{dec},\mathrm{ch}1}K_{i}^{\mathrm{eq},\mathrm{ch}1}}{k_{i}^{\mathrm{dec}}+k_{i}^{\mathrm{stab}}\left[M\right]\;}\left[66\right]\left[8\right]+\frac{k_{i}^{\mathrm{dec},\mathrm{ch}2}K_{i}^{\mathrm{eq},\mathrm{ch}2}}{k_{i}^{\mathrm{dec}}+k_{i}^{\mathrm{stab}}\left[M\right]\;}\left[68\right]\left[6\right]$$

Note that these are microcanonical equilibrium constants that do not include Boltzmann average over the states of ozone, but instead are introduced for every individual state of a product at given energy $E_{i}$ of a resonance:

(30) $$K_{i}^{\mathrm{eq},\mathrm{ch}1}=\frac{\left(2J+1\right)\;e^{-E_{i}/kT}}{Q_{\mathrm{ch}1}\;e^{-\Delta \mathrm{ZPE}/kT}}$$

(31) $$K_{i}^{\mathrm{eq},\mathrm{ch}2}=\frac{\left(2J+1\right)\;e^{-E_{i}/kT}}{Q_{\mathrm{ch}2}\;}$$

**Figure 2:**
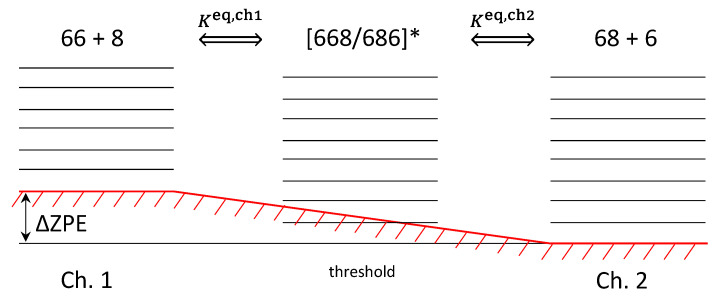
Two distinct channels of ozone formation (left and right) and the metastable ozone states (middle) in the case of single isotopic substitution. Here “6” denotes ^16^O whereas “8” denotes ^18^O. Lower energy channel corresponds to the heavier diatomic reagent ^16^O^18^O with smaller zero-point energy. Upper channel corresponds to the lighter diatomic reagent ^16^O^16^O. Energy difference of the two channels, ΔZPE, is indicated.

Calculations of the reagent partition functions for two channels, $Q_{\mathrm{ch}1}$ and $Q_{\mathrm{ch}2}$, are standard. The factor 2J+1 accounts for space degeneracy, related to the quantum number $M_{J}$. One important element to discuss is the factor $e^{-\Delta \mathrm{ZPE}/kT}$ in the denominator of the first of these expressions. Figure 2 is used to emphasize that the two reaction channels are not entirely isoergic. 

Although the electronic energies of the reagents in the two channels are exactly the same, their vibrational zero-point energies are different. For example, in the singly substituted case, lower energy channel corresponds to the heavier diatomic ^16^O^18^O, which defines threshold of the recombination process. Higher energy channel corresponds to the lighter diatomic ^16^O^16^O. The difference of their vibrational zero-point energies is defined as (for single substitution):

$$\Delta \mathrm{ZPE}=\mathrm{ZPE}\left(66\right)-\mathrm{ZPE}\left(68\right)=22.27\;{\mathrm{cm}}^{-1}$$

Qualitatively, one may say that in the case of single isotopic substitution the Channel 1 is lifted above threshold, defined by the Channel 2, as shown in Figure 2. This energy difference, called ΔZPE, enters our formalism through the expressions for the equilibrium constant of Channel 1, Equation (30). 

Putting together Equations (13), (21), (22), (29) and (31), we obtain:

(32) $$R_{\mathrm{sym}\;\;}=\;\left[M\right]\left[68\right]\left[6\right]\frac{k^{\mathrm{stab}}}{Q_{\mathrm{ch}2}}\underset{i}{\sum }\left(2J+1\right)p_{i}^{\mathrm{sym}}\frac{\;\frac{\Gamma_{i}^{\mathrm{ch}2}}{\hbar }}{\frac{\Gamma_{i}^{\mathrm{tot}}}{\hbar }+k_{i}^{\mathrm{stab}}\left[M\right]}e^{-\frac{E_{i}}{kT}}\;+\;\left[M\right]\left[66\right]\left[8\right]\frac{k^{\mathrm{stab}}\;\;}{Q_{\mathrm{ch}1}}\underset{i}{\sum }\left(2J+1\right)p_{i}^{\mathrm{sym}}\frac{\;\frac{\Gamma_{i}^{\mathrm{ch}1}\;}{\hbar }}{\frac{\Gamma_{i}^{\mathrm{tot}}}{\hbar }+k_{i}^{\mathrm{stab}}\left[M\right]}e^{-\frac{E_{i}-\Delta \mathrm{ZPE}}{kT}}$$

(33) $$R_{\mathrm{asym}\;}=\left[M\right]\left[68\right]\left[6\right]\;\frac{k^{\mathrm{stab}}\;\;}{Q_{\mathrm{ch}2}}\underset{i}{\sum }\left(2J+1\right)p_{i}^{\mathrm{asym}}\frac{\;\frac{\Gamma_{i}^{\mathrm{ch}2}}{\hbar }}{\frac{\Gamma_{i}^{\mathrm{tot}}}{\hbar }+k_{i}^{\mathrm{stab}}\left[M\right]}\;e^{-\frac{E_{i}}{kT}}\;+\;\left[M\right]\left[66\right]\left[8\right]\;\frac{k^{\mathrm{stab}}\;\;}{Q_{\mathrm{ch}1}}\underset{i}{\sum }\left(2J+1\right)p_{i}^{\mathrm{asym}}\frac{\;\frac{\Gamma_{i}^{\mathrm{ch}1}}{\hbar }}{\frac{\Gamma_{i}^{\mathrm{tot}}}{\hbar }+k_{i}^{\mathrm{stab}}\left[M\right]}e^{-\frac{E_{i}-\Delta \mathrm{ZPE}}{kT}}\;$$

Note that the term $e^{-\Delta \mathrm{ZPE}/kT}$ was moved to the numerator, to appear together with $\Gamma_{i}^{\mathrm{ch}1}/\hbar$. The reason behind this move is that the decay of resonances through Channel 1, given by $\Gamma_{i}^{\mathrm{ch}1}/\hbar$, is efficient only at energies above threshold for this channel, which is ΔZPE (see Figure 2). Therefore, it makes sense to measure energies of such resonances relative to the threshold of this channel, writing the Boltzmann factor as $e^{-(E_{i}-\Delta \mathrm{ZPE})/kT}$. 

At this point it becomes clear that the two terms in Equation (32) for *R*_sym_ correspond to $R_{S}$ and $R_{I}$ whereas the two terms in Equation (33) for *R*_asym_ correspond to $R_{A}$ and $R_{B}$:

(34) $$R_{\mathrm{sym}\;}=\;R_{S\;\;}+\;R_{I\;}$$

(35) $$R_{\mathrm{asym}\;\;}=\;R_{A\;\;}+\;R_{B\;}$$

This is because $R_{S}$ and $R_{A}$ correspond to the reagents in Channel 2, whereas $R_{I}$ and $R_{B}$ correspond to the reagents in Channel 1. Therefore, the rates of recombination through four pathways are introduced as:

(36) $$R_{A}\equiv \kappa_{A}\left[M\right]\left[68\right]\left[6\right]$$

(37) $$R_{B}\equiv \kappa_{B}\left[M\right]\left[66\right]\left[8\right]$$

(38) $$R_{S}\equiv \;\kappa_{S}\left[M\right]\left[68\right]\left[6\right]$$

(39) $$R_{I}\equiv \kappa_{I}\left[M\right]\left[66\right]\left[8\right]$$

However, based on Equations (32) and (33), the expressions for $\kappa_{A}$*,* $\kappa_{B}$*,* $\kappa_{S}$ and $\kappa_{I}$ would come out bulky. In order to make them more intuitive, we will introduce into the formalism several handy moieties.

### 2.2. Kinetic Weight of a Resonance and the Dynamical Partition Function

The values of ratios in Equations (32) and (33) depend on the relative efficiencies of resonance formation, decay and stabilization. It is convenient to name this moiety the kinetic weight of a resonance, or simply the *weight*. If there would be only one channel of resonance formation/decay, the expression for the weight would be:

(40) $$w_{i}=\frac{\;\frac{\Gamma_{i}}{\hbar }}{\frac{\Gamma_{i}}{\hbar }+k_{i}^{\mathrm{stab}}\left[M\right]}$$

In the low-pressure limit, $k_{i}^{\mathrm{stab}}\left[M\right]\ll \Gamma_{i}/\hbar$, the weight reaches its maximum value, $w_{i}\to 1$, and the role of a resonance in the recombination process is maximized. In contrast, in the high-pressure limit, $k_{i}^{\mathrm{stab}}\left[M\right]\gg \Gamma_{i}/\hbar$, the weight is $w_{i}\to 0$, and the contribution of a resonance may be small. At given finite pressure, different resonances have different weights, determined by their widths $\Gamma_{i}$. Broader resonances have larger weights, while narrower resonances have lower weights. One can consider a limiting case of a bound state with zero width, $\Gamma_{i}=0$. Such states would have $w_{i}=0$ at any pressure. This makes sense, since the bound states do not decay spontaneously and are not formed directly from the reagents, making no contribution to the recombination process. 

When multiple (in our case two) decay channels are present, one can still use Equation (40) to define the weight, with the total width $\Gamma_{i}^{\mathrm{tot}}$ instead of $\Gamma_{i}$. This can be introduced into Equations (32) and (33) by factoring out the channel specific ratios $\Gamma_{i}^{\mathrm{ch}1}/\Gamma_{i}^{\mathrm{tot}}$ and $\Gamma_{i}^{\mathrm{ch}2}/\Gamma_{i}^{\mathrm{tot}}$ as we will demonstrate further below.

Note that summation in Equations (32) and (33) goes over the metastable states of ozone, multiplied by Boltzmann factors and degeneracy factors, and thus very much resembles a partition function at the transition state, $Q^{‡}$, used in the RRKM theory [34], except that our formulae take into account the processes of formation, decay and stabilization of scattering resonances, and therefore are more general. Inspired by this similarity, we found it instructive to introduce the *dynamical partition function*, $\tilde{Q}$, for four pathways of ozone formation, defined as follows:

(41) $${\tilde{Q}}_{A}\;\equiv \;\underset{i}{\sum }\left(2J+1\right)\;w_{i}\frac{\;\Gamma_{i}^{\mathrm{ch}2}}{\;\Gamma_{i}^{\mathrm{tot}}}\;p_{i}^{\mathrm{asym}}\;e^{-\frac{E_{i}}{kT}}$$

(42) $${\tilde{Q}}_{B}\;\equiv \;\underset{i}{\sum }\left(2J+1\right)\;w_{i}\frac{\;\Gamma_{i}^{\mathrm{ch}1}}{\;\Gamma_{i}^{\mathrm{tot}}}\;p_{i}^{\mathrm{asym}}\;e^{-\frac{E_{i}-\Delta \mathrm{ZPE}}{kT}}$$

(43) $${\tilde{Q}}_{S}\;\equiv \;\underset{i}{\sum }\left(2J+1\right)\;w_{i}\frac{\;\Gamma_{i}^{\mathrm{ch}2}}{\;\Gamma_{i}^{\mathrm{tot}}}\;p_{i}^{\mathrm{sym}}\;\;e^{-\frac{E_{i}}{kT}}$$

(44) $${\tilde{Q}}_{I}\;\equiv \;\underset{i}{\sum }\left(2J+1\right)\;w_{i}\frac{\;\Gamma_{i}^{\mathrm{ch}1}}{\;\Gamma_{i}^{\mathrm{tot}}}\;p_{i}^{\mathrm{sym}}\;e^{-\frac{E_{i}-\Delta \mathrm{ZPE}}{kT}}$$

Note that each factor in the definition of the dynamical partition function $\tilde{Q}$ is unitless: the degeneracy, the weight of a resonance (determined by the pressure of bath gas and by the total resonance width, see Equation (40) above), stabilization probability, and the Boltzmann factor (including the ΔZPE shift for pathways *B* and *I* that originate in the upper Channel 1, see Figure 2). The values of $\tilde{Q}$ for four pathways are obtained from four combinations of the relative decay rates through one of two reagent channels (either $\Gamma_{i}^{\mathrm{ch}1}/\Gamma_{i}^{\mathrm{tot}}$ or $\Gamma_{i}^{\mathrm{ch}2}/\Gamma_{i}^{\mathrm{tot}}$) with two stabilization probabilities (either $p_{i}^{\mathrm{sym}}$ or $p_{i}^{\mathrm{asym}}$). 

It is also important to note that our definition of $\tilde{Q}$ takes into account the probabilities of wave function in the inner region of the PES (either $p_{i}^{\mathrm{sym}}$ or $p_{i}^{\mathrm{asym}}$), where the resonance can be quenched into a stable ozone molecule by collisions with bath gas. This factor plays important role in obtaining both physically meaningful and numerically converged value of $\tilde{Q}.$ As we will discuss in Section 3.3 below, the spectrum of states above dissociation threshold contains, unavoidably, the free-particle states with large widths (short lifetimes). Since they possess large weights ($w_{i}\;\sim \;1$) one may think that they would make large contributions to the recombination process, which would be unphysical. This is where the probabilities $p_{i}^{\mathrm{sym}}$ and $p_{i}^{\mathrm{asym}}$ come into play. The free-particle states, hosted by the asymptotic part of the PES, have $p_{i}^{\mathrm{sym}}$ and $p_{i}^{\mathrm{asym}}$ close to zero, which makes their contributions to $\tilde{Q}$ vanishingly small, as it should be. So, in order to contribute to the dynamical partition function $\tilde{Q}$ and the recombination reaction, a resonance should possess non-vanishing weight $w_{i}$ and probability (either $p_{i}^{\mathrm{sym}}$ or $p_{i}^{\mathrm{asym}}$), both. A very narrow resonance has $p_{i}^{\mathrm{sym}}$ or $p_{i}^{\mathrm{asym}}$ close to one, but small weight $w_{i}$. A very broad resonance has large weight $w_{i}$ but small probabilities $p_{i}^{\mathrm{sym}}$ and $p_{i}^{\mathrm{asym}}$. Neither makes large contribution to $\tilde{Q}$. Only the resonances with optimal combination of the weight and probability play role in the recombination process. 

With these definitions, the four pathway-specific rate coefficients are concisely written as follows:

(45) $$\kappa_{A}\equiv k^{\mathrm{stab}}\frac{{\tilde{Q}}_{A}}{Q_{\mathrm{ch}2}}$$

(46) $$\kappa_{B}\equiv k^{\mathrm{stab}}\frac{{\tilde{Q}}_{B}}{Q_{\mathrm{ch}1}}$$

(47) $$\kappa_{S}\;\equiv k^{\mathrm{stab}}\frac{{\tilde{Q}}_{S}\;}{Q_{\mathrm{ch}2}}$$

(48) $$\kappa_{I}\;\equiv k^{\mathrm{stab}}\frac{{\tilde{Q}}_{I}}{Q_{\mathrm{ch}1}}$$

Note that each of these formulae has a simple intuitive form: recombination rate coefficient is a product of stabilization rate coefficient with the ratio of the partition functions (products over reagents). All complexity is absorbed by the dynamical partition functions of four recombination pathways, computed quantum mechanically: ${\tilde{Q}}_{A}$, ${\tilde{Q}}_{B}$, ${\tilde{Q}}_{S}$, ${\tilde{Q}}_{I}$.

### 2.3. Formation of Symmetric and Asymmetric Molecules

One has to realize that both *R*_sym_ and *R*_asym_ include contributions from two reaction channels with different reagents [66][8], in Channel 1 and [68][6], in Channel 2, see Equations (49) and (50). Therefore, rate coefficient *κ*_sym_ is not a simple sum of *κ*_S_ and *κ*_I_, and likewise *κ*_asym_ is not a simple sum of *κ*_A_ and *κ*_B_. The following manipulations, however, permit to derive all necessary formulae:

(49) $$R_{\mathrm{sym}}=\;R_{S\;\;}+\;R_{I\;\;}=\;\kappa_{S}\left[M\right]\left[68\right]\left[6\right]\;+\;\kappa_{I}\left[M\right]\left[66\right]\left[8\right]\;\;=\;\left(\kappa_{S}+\kappa_{I}\frac{\left[66\right]\left[8\right]}{\left[68\right]\left[6\right]}\right)\left[68\right]\left[6\right]\left[M\right]=\kappa_{\mathrm{sym}}\left[68\right]\left[6\right]\left[M\right]$$

(50) $$R_{\mathrm{asym}}=\;R_{A\;\;}+\;R_{B\;\;}=\;\kappa_{A}\left[M\right]\left[68\right]\left[6\right]\;+\;\kappa_{B}\left[M\right]\left[66\right]\left[8\right]\;\;=\;\left(\kappa_{A}+\kappa_{B}\frac{\left[66\right]\left[8\right]}{\left[68\right]\left[6\right]}\right)\left[68\right]\left[6\right]\left[M\right]=\kappa_{\mathrm{asym}}\left[68\right]\left[6\right]\left[M\right]$$

In this approach the contributions of two reaction channels are added together, by re-expressing the two rates through the concentrations of reagents in the lowest energy channel (Channel 2 in the singly substituted case). 

Note that the ratio of four concentrations in these expressions represents the equilibrium constant for isotope exchange between the two channels, with forward direction defined to be from Channel 2 to Channel 1:

(51)  68+6 ⇌66+8

(52) $$K^{\mathrm{ex}}\;=\;\frac{\left[66\right]\left[8\right]}{\left[68\right]\left[6\right]}\;=\frac{Q_{\mathrm{ch}1}e^{-\Delta \mathrm{ZPE}/kT}}{Q_{\mathrm{ch}2}}.\;$$

The value of this equilibrium constant is expected to be on the order of 1/2 due to symmetry of the homonuclear diatomic reagent in the Channel 1, in which every other rotational state is forbidden by quantum mechanical selection rules.

With this definition, the expressions for product-specific recombination rate coefficients are:

(53) $$\kappa_{\mathrm{sym}}\;\equiv \;\kappa_{S}+\kappa_{I}K^{\mathrm{ex}}$$

(54) $$\kappa_{\mathrm{asym}}\;\equiv \;\kappa_{A}+\kappa_{B}K^{\mathrm{ex}}$$

Indeed, neither of these expressions is a simple sum. The isotope exchange constant $K^{\mathrm{ex}}$ enters these expressions, multiplying the rate constant for the upper channel. 

Substitution of Equations (45)–(48) for four rate constants into Equations (53) and (54) permits to obtain the following expressions:

(55) $$\kappa_{\mathrm{sym}}\;\equiv \;k^{\mathrm{stab}}\frac{{\tilde{Q}}_{\mathrm{sym}}\;}{Q_{\mathrm{ch}2}}$$

(56) $$\kappa_{\mathrm{asym}}\;\equiv \;k^{\mathrm{stab}}\frac{{\tilde{Q}}_{\mathrm{asym}}\;}{Q_{\mathrm{ch}2}}$$

where we introduced the dynamical partition functions for the formation of symmetric and asymmetric ozone molecules as:

(57) $${\tilde{Q}}_{\mathrm{sym}}\;\equiv \;{\tilde{Q}}_{S}+{\tilde{Q}}_{I}e^{-\frac{\;\Delta \mathrm{ZPE}}{kT}}$$

(58) $${\tilde{Q}}_{\mathrm{asym}}\;\equiv \;{\tilde{Q}}_{A}+{\tilde{Q}}_{B}e^{-\frac{\;\Delta \mathrm{ZPE}}{kT}}$$

Note that $\kappa_{\mathrm{sym}}$ and $\kappa_{\mathrm{asym}}$ use the same reference channel, namely, the lower energy Channel 2, so the partition function of this channel, $Q_{\mathrm{ch}2}$, shows up in the denominator of both expressions. Also note, that in ${\tilde{Q}}_{\mathrm{sym}}$ or ${\tilde{Q}}_{\mathrm{asym}}$ the two contributions come not as a simple sum; the contribution of the upper channel is multiplied by $e^{-\Delta \mathrm{ZPE}/kT}$.

It is instructive to express ${\tilde{Q}}_{\mathrm{sym}}$ and ${\tilde{Q}}_{\mathrm{asym}}$ through the properties of individual resonances. Substitution of Equations (41)–(44) into (57) and (58) gives us:

(59) $${\tilde{Q}}_{\mathrm{sym}}=\underset{i}{\sum }\left(2J+1\right)\;w_{i}\frac{\;\Gamma_{i}^{\mathrm{ch}2}}{\;\Gamma_{i}^{\mathrm{tot}}}\;p_{i}^{\mathrm{sym}}e^{-\frac{E_{i}}{kT}}\;+\;e^{-\frac{\;\Delta \mathrm{ZPE}}{kT}}\underset{i}{\sum }\left(2J+1\right)\;w_{i}\;\frac{\;\Gamma_{i}^{\mathrm{ch}1}}{\;\Gamma_{i}^{\mathrm{tot}}}\;p_{i}^{\mathrm{sym}}e^{-\frac{E_{i}\;-\;\Delta \mathrm{ZPE}}{kT}}\;=\underset{i}{\sum }\left(2J+1\right)\;w_{i}\;\;p_{i}^{\mathrm{sym}}\;e^{-\frac{E_{i}}{kT}};$$

(60) $${\tilde{Q}}_{\mathrm{asym}}=\;\underset{i}{\sum }\left(2J+1\right)\;w_{i}\;\frac{\;\Gamma_{i}^{\mathrm{ch}2}}{\;\Gamma_{i}^{\mathrm{tot}}}\;p_{i}^{\mathrm{asym}}e^{-\frac{E_{i}}{kT}}\;+\;e^{-\frac{\;\Delta \mathrm{ZPE}}{kT}}\underset{i}{\sum }\left(2J+1\right)\;w_{i}\;\frac{\;\Gamma_{i}^{\mathrm{ch}1}}{\Gamma_{i}^{\mathrm{tot}}}\;\;p_{i}^{\mathrm{asym}}e^{-\frac{E_{i}\;-\;\Delta \mathrm{ZPE}}{kT}}\;=\;\underset{i}{\sum }\left(2J+1\right)\;w_{i}\;\;p_{i}^{\mathrm{asym}}\;e^{-\frac{E_{i}}{kT}}.$$

This result is very interesting, since several moieties disappeared in the final expressions for ${\tilde{Q}}_{\mathrm{sym}}$ and ${\tilde{Q}}_{\mathrm{asym}}$. First, all ΔZPE factors canceled analytically, which means that quantum zero-point energies are unlikely to cause any isotope effect related to symmetric vs. asymmetric ozone molecules (at least not directly). Then, two contributions to the total resonance width, $\Gamma_{i}^{\mathrm{ch}1}$ and $\Gamma_{i}^{\mathrm{ch}2}$, added up and cancelled with $\Gamma_{i}^{\mathrm{tot}}$. This means that, for the description of symmetry driven isotope effects, the widths of scattering resonances, or lifetimes of the metastable states, may not be important either (again, not directly). They still enter the weights $w_{i}$, but the same weights enter ${\tilde{Q}}_{\mathrm{sym}}$ and ${\tilde{Q}}_{\mathrm{asym}}$. The only difference between ${\tilde{Q}}_{\mathrm{sym}}$ and ${\tilde{Q}}_{\mathrm{asym}}$ comes from the stabilization probabilities, $\;p_{i}^{\mathrm{sym}}$ vs. $\;p_{i}^{\mathrm{asym}}$. Importantly, in the case of double isotopic substitution the formulae identical to Equations (59) and (60) are obtained, as we show in the Supplementary Materials.

## 3. Results and Discussion

### 3.1. Pathway-Specific Rate Coefficients

Our database of the rovibrational states of ozone [22] includes 380,434 states of singly substituted isotopologue and 400,183 states of doubly substituted isotopologue, at energies up to 800 cm^−1^ above dissociation threshold, computed for the values of total angular momentum of the molecule from *J* = 4 to *J* = 56 with a step of 4, within the symmetric-top rotor approximation for the values of *K* from zero to *K = J*, with a step of 2. Out of these states, about 520,000 states are the bound states below dissociation threshold of singly and doubly substituted isotopologues, whereas the remaining 260,000 states are scattering resonances above threshold. The approach developed above uses properties of all these scattering resonances as input parameters ($E_{i}$, $\Gamma_{i}^{\mathrm{ch}1}$, $\Gamma_{i}^{\mathrm{ch}2}$, $p_{i}^{\mathrm{sym}}$, $p_{i}^{\mathrm{asym}}$ for each state), to compute the relevant dynamical partition functions (${\tilde{Q}}_{A}$*,* ${\tilde{Q}}_{B}$*,* ${\tilde{Q}}_{S}$, ${\tilde{Q}}_{I}$) by summation over these states, see Equations (41)–(44), and to predict the corresponding recombination rate coefficients (*κ*_A_*,* *κ*_B_*,* *κ*_S_, *κ*_I_) using Equations (45)–(48). 

Theoretically predicted rate coefficients for the four pathways in singly and doubly substituted ozone molecules are added to the Table 1. The most significant new addition to the previous work is theoretical prediction of rate coefficients for the insertion pathways, *κ*_I_, never computed before by anyone else. Experimental information for the insertion pathway in ozone is also rather limited and consists of only two values of rate coefficient *κ*_I_ cited in Table 1, both at room temperature, one for the insertion of 6 into 88, and the other for the insertion of 8 into 66. In agreement with experiment, our theory predicts very small rate coefficients for the insertion pathway, in the range of two orders of magnitude smaller than rate coefficients for the other three pathways (compare to *κ*_A_*,* *κ*_B_ and *κ*_S_ in Table 1). This is consistent with an *indirect* reaction path, that must circle the origin in Figure 1, going from the reagent channel at the bottom of the figure (Channel 1 with a homonuclear diatomic) into the product well at the top of the figure (to form a symmetric ozone molecule). A schematic of this process is provided in the Supplementary Materials, Figure S2. Absolute value of theoretically predicted *κ*_I_ is quite close to the experimental value in the case of double isotopic substitution (see Table 1), whereas it deviates more in the case of single isotopic substitution. One must remember, however, that dealing with the insertion pathway we look at the relatively small rate coefficients influenced by both theoretical assumptions and experimental limitations.

Also, in agreement with experiment, our results indicate that insertion is much slower in the singly substituted case, Equation (11), compared to the doubly substituted case, Equation (12). In order to quantify this phenomenon, we propose to introduce the ratio of corresponding rate coefficients, namely:

(61) $$\xi =\frac{\kappa_{I}^{\left(d\right)}}{\kappa_{I}^{\left(s\right)}}$$

where indexes (s) and (d) stand for single and double isotopic substitutions, respectively. In the experiment this ratio is larger than one, close to the factor of five, ξ = 4.8. Importantly, our theory shows the same effect (ξ > 1) with even larger magnitude, ξ = 7.9 (see Table 1). 

The difference of experimental and theoretical absolute values of ξ-effect may originate in the approximate theoretical treatment of stabilization step (since we assume the same value of stabilization cross section for single and double isotopic substitutions, while they may be different in reality), or in the details of potential energy surface (since this indirect reaction path crosses several transition states, and thus is expected to be sensitive to the reaction landscape), or in the difficulties of experimental measurements (since this is a minor pathway with very small rate coefficient). Despite of these multiple uncertainties, a semiquantitative agreement of theory with experiment is obtained, which is already a successful first step.

An important qualitative question is why the insertion is much faster in the doubly substituted case, $6+88\;\overset{\;\kappa_{I}\;}{\to }\;868$, compared to the singly substituted case, $8+66\;\overset{\;\kappa_{I}\;}{\to }\;686$. For this, let’s consider the effective mass of reagents in these two processes. It may be counterintuitive at first, but it appears that the reduced mass of reagents in the doubly substituted case:

(62) $$\mu^{\left(d\right)}=\;\frac{16\times 36}{16+36}\approx 11.07\;\left(\mathrm{amu}\right)$$

is appreciably smaller than in the singly substituted case:

(63) $$\mu^{\left(s\right)}=\;\frac{18\times 32}{18+32}\approx 11.52\;\left(\mathrm{amu}\right)$$

Smaller effective mass is associated with faster motion and thus larger rate coefficients at the same temperature, but also with more efficient tunneling. Since the rate coefficients are small for the insertion, the effect of tunneling becomes comparable [22]. The “shape-type” resonances are populated by tunneling, which is exponentially sensitive to the masses of isotopes. It should also be stressed that, in the case of single substitution, Channel 1 (where the insertion process starts) is the upper channel, but in the case of double substitution Channel 1 is the lower channel. All experimental data indicate that lower channels exhibit higher recombination rates than upper channels (see discussion of the ΔZPE effect below), and it looks like the insertion pathway follows the same rule.

As for other three recombination pathways included in Table 1, the values of rate coefficients *κ*_A_ and *κ*_S_ obtained here are very similar to those reported in the earlier work by Teplukhin et al. [22], while the value of *κ*_B_ is different. This is because a bug was discovered in the original version of the code, due to which the reduced mass of ^16^O^16^O (instead of ^18^O^18^O) was used erroneously for the calculations of the reagent partition function for Channel 1 in the doubly substituted case. This problem was corrected in the updated version of the code, and resulted in a significant change of *κ*_B_ and $K^{\mathrm{ex}}$ for the doubly substituted case (but no significant change in the isotope effects, where these two moieties enter together and the effect of diatomic mass cancels, see below).

Overall, the trends we see for the values of *κ*_A_*,* *κ*_B_*,* *κ*_S_ and *κ*_I_ computed theoretically for single and double substitutions of ^18^O in ozone follow experimental results reasonably well. Namely, the values of 2*κ*_S_ are very similar in the singly and doubly substituted cases, and their magnitudes are always in between of those for 2*κ*_A_ and *κ*_B_, whereas the order of 2*κ*_A_ and *κ*_B_ is opposite in the singly and doubly substituted cases. Namely, in the singly substituted case 2*κ*_A_ > *κ*_B_ whereas in the doubly substituted case 2*κ*_A_ < *κ*_B_. This is explained by the fact that in the singly substituted case the lower energy pathway is *A* (since ZPE of ^16^O^18^O is smaller than that of ^16^O^16^O, see Figure 2 above), but in the doubly substituted case the lower energy pathway is *B* (since ZPE of ^16^O^18^O is larger than that of ^18^O^18^O, see Figure S1 in the Supplementary Materials). The mechanism of this ΔZPE phenomenon was discussed in detail in the literature [14,15,16,17,20,21,22], and will not be reiterated here. 

### 3.2. Isotope Effects

The most convenient and reliable characteristic of ozone formation process is the value of isotope effect computed as a *ratio* of pathway-specific rate coefficients, since in the ratio some of uncertain parameters cancel. This was the case in the experimental studies of ozone [1,3,4,6], and is also true for our theory. Namely, the theory developed above relies on a simple empirical stabilization model with one value of stabilization cross section σ_stab_ determined approximately in a simplified semiclassical study [30]. But, if one considers the ratio of rate coefficients, then the values of stabilization rate coefficients cancel, enabling more reliable predictions and more straightforward comparison with experiment. 

Thus, the symmetry-driven *η*-effect in the singly substituted case can be characterized by the ratio:

(64) $$\eta^{\left(s\right)}\;=\;\frac{\kappa_{\mathrm{asym}}}{2\kappa_{\mathrm{sym}}}=\;\frac{\kappa_{A}+\kappa_{B}K^{\mathrm{ex}}}{2\left(\kappa_{S}\;+\kappa_{I}K^{\mathrm{ex}}\right)}=\;\frac{{\tilde{Q}}_{A}+{\tilde{Q}}_{B}e^{-\frac{\;\Delta \mathrm{ZPE}}{kT}}\;}{2\left({\tilde{Q}}_{S}+{\tilde{Q}}_{I}e^{-\frac{\;\Delta \mathrm{ZPE}}{kT}}\right)}\;=\;\frac{{\tilde{Q}}_{\mathrm{asym}}\;}{2{\tilde{Q}}_{\mathrm{sym}}}$$

We see that the final expression for *η* includes only the dynamical partition functions $\tilde{Q}$, computed rigorously using quantum mechanics. Similar, in the case of double substitution (see Supplementary Materials) we obtain:

(65) $$\eta^{\left(d\right)}\;=\;\frac{\kappa_{\mathrm{asym}}\;}{2\kappa_{\mathrm{sym}}}=\frac{\kappa_{A}K^{\mathrm{ex}}\;+\kappa_{B}}{2\left(\kappa_{S}K^{\mathrm{ex}}\;+\kappa_{I}\right)}=\;\frac{{\tilde{Q}}_{A}e^{-\frac{\;\Delta \mathrm{ZPE}}{kT}}+{\tilde{Q}}_{B}}{2\left({\tilde{Q}}_{S}e^{-\frac{\;\Delta \mathrm{ZPE}}{kT}}+{\tilde{Q}}_{I}\right)}\;=\frac{{\tilde{Q}}_{\mathrm{asym}}}{2{\tilde{Q}}_{\mathrm{sym}}}$$

i.e., the same final result. 

Next, two terms in the numerator of Equations (64) and (65) can be used to set up the ratio for characterization of the ΔZPE-driven ζ-effect. For single substitution, from Equation (64):

(66) $$\zeta^{\left(s\right)}=\frac{\kappa_{A}}{\kappa_{B}K^{\mathrm{ex}}}=\frac{{\tilde{Q}}_{A}}{{\tilde{Q}}_{B}e^{-\frac{\;\Delta \mathrm{ZPE}}{kT}}}$$

and similar for double substitution, from Equation (65):

(67) $$\zeta^{\left(d\right)}=\frac{\kappa_{B}}{\kappa_{A}K^{\mathrm{ex}}}=\frac{{\tilde{Q}}_{B}}{{\tilde{Q}}_{A}e^{-\frac{\;\Delta \mathrm{ZPE}}{kT}}}$$

Note that all definitions are set such that no anomalous isotope effect would correspond to ζ = 1 and *η* = 1 in all cases, and all experimentally observed isotope effects would correspond to ζ > 1 and *η* > 1. 

Equations (64)–(67) were used to populate Table 1 with experimental and theoretical values of parameters ζ and *η* for singly and doubly substituted cases. Theoretically predicted ζ-effect is large and goes in the same direction as in the experiment (ζ > 1) in both singly and doubly substituted cases, although in our calculations its magnitude, about ζ = 1.42 ± 0.03 (in the singly and doubly substituted cases, respectively), is somewhat smaller compared to the experiment, which is ζ = 1.73 ± 0.04 (in the singly and doubly substituted cases). Concerning the mysterious *η*-effect driven by symmetry, our calculations give *η* = 1.02 and 1.08 (in the singly and doubly substituted cases), which is significantly smaller than the experimental values of *η* = 1.16 ± 0.03. The deviation is largest in the case of single substitution, which is particularly sad since this case is relevant to the processes in Earth’s atmosphere, with low abundance of ^18^O. The direction of all isotope effects in Table 1 is, nevertheless, consistent with experiment (*η* > 1 in all cases). 

It was proposed in the literature [10,11,12,13] that *η*-effect is exclusively driven by symmetry (somehow), whereas the ΔZPE difference of two formation channels manifests only in ζ-effect. The logic for this statement can be seen in Equations (64) and (65), where the ΔZPE-factor cancels out, in contrast to Equations (66) and (67), where it survives. While here we came to the same analytic result, our interpretation of the origin of *η*-effect is different. Energies and widths of scattering resonances near the process threshold are strongly affected by the presence of ΔZPE “step” on the potential energy landscape of the process, and even if the Boltzmann factor $e^{-\Delta \mathrm{ZPE}/kT}$ cancels out analytically in the Equations (66) and (67), the values of ${\tilde{Q}}_{\mathrm{sym}}$ and ${\tilde{Q}}_{\mathrm{asym}}$ in these formulae still carry information about the spectra of scattering resonances, influenced by the ΔZPE. To this extent, the small *η*-effect seen in our results is also related to the ΔZPE. 

### 3.3. Distribution of Resonance Widths

Although all scattering resonances are included in the dynamical partition functions $\tilde{Q}$ introduced above, different resonances exhibit different properties and those vary dramatically through the database. Therefore, different resonances make different contributions to the rate of the reaction, and to the resultant isotope effects. Here we will take closer look at the distribution of resonance widths $\Gamma_{i}$ that determine decay rates (inversely proportional to their lifetimes). For this purpose, it is convenient to consider zero-pressure limit, when:

(68) $${\tilde{Q}}_{\mathrm{sym}}^{\circ }=\underset{i}{\sum }\left(2J+1\right)\;p_{i}^{\mathrm{sym}}e^{-\frac{E_{i}}{kT}}$$

(69) $${\tilde{Q}}_{\mathrm{asym}}^{\circ }=\underset{i}{\sum }\left(2J+1\right)\;p_{i}^{\mathrm{asym}}e^{-\frac{E_{i}}{kT}}$$

and therefore:

(70) $${\tilde{Q}}_{\mathrm{tot}}^{\circ }\;=\underset{i}{\sum }\left(2J+1\right)\;p_{i}^{\mathrm{tot}}\;e^{-\frac{E_{i}}{kT}}$$

The point is that in the zero-pressure limit all resonances are equally weighted, with $w_{i}=1$ regardless of the width $\Gamma_{i}$, and therefore all resonances are made “visible” in the distribution. Moreover, if we set $p_{i}^{\mathrm{tot}}=1$ (see below) then the expression for the dynamical partition function ${\tilde{Q}}_{\mathrm{tot}}^{\circ }$ gives just the Boltzmann average over scattering resonances including rotational degeneracies, equivalent to the statistical partition function at the transition state, $Q^{‡}$. 

**Figure 3:**
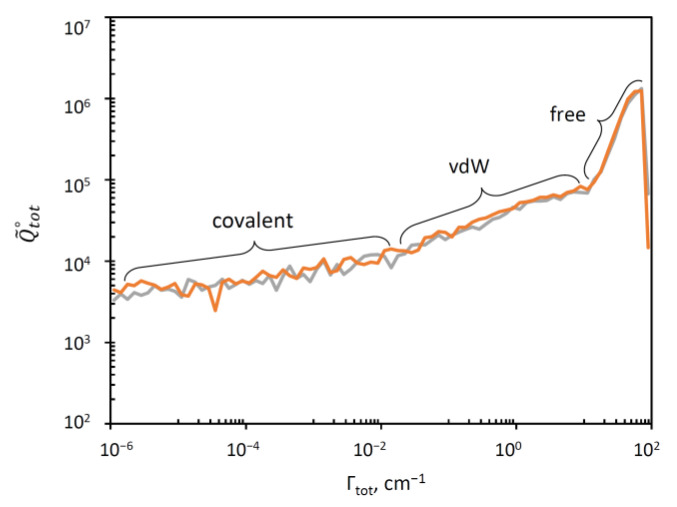
Distribution of resonance widths in singly (**grey**) and doubly (**orange**) substituted ozone molecules. Three ranges with different trends can be identified.

In Figure 3 we plotted a “raster” image of ${\tilde{Q}}_{\mathrm{tot}}^{\circ }$ versus Γ (or vs. $\Gamma_{i}^{\mathrm{tot}}$ to be exact) obtained as a histogram with boxes generated using a log-scale for Γ. This can be thought of as ${\tilde{Q}}_{\mathrm{tot}}^{\circ }$ stretched along the Γ-axis (such that the sum of values in the histogram boxes gives the total value of ${\tilde{Q}}_{\mathrm{tot}}^{\circ }$ in the equation above). The range of resonance widths in this picture is eight-orders-of-magnitude broad, from the extremely narrow (Γ ~ 10^−6^ cm^−1^) to very broad resonances (Γ ~ 100 cm^−1^). The data for singly and doubly substituted molecules are presented in Figure 3 separately, but they look very similar and both indicate that the overall distribution is likely to represent a convolution of three components that dominate in three different ranges of Γ. 

Fortunately, our theory gives an efficient, simple and rigorous method for “deconvolution” of this distribution, by splitting the total probability, and the total partition function onto three contributions:

$$p_{i}^{\mathrm{tot}}=1=\;p_{i}^{\mathrm{cov}}+\;p_{i}^{\mathrm{vdw}}+p_{i}^{\mathrm{free}}$$

$${\tilde{Q}}_{\mathrm{tot}}^{\circ }={\tilde{Q}}_{\mathrm{cov}}^{\circ }+{\tilde{Q}}_{\mathrm{vdw}}^{\circ }+{\tilde{Q}}_{\mathrm{free}}^{\circ }$$

**Figure 4:**
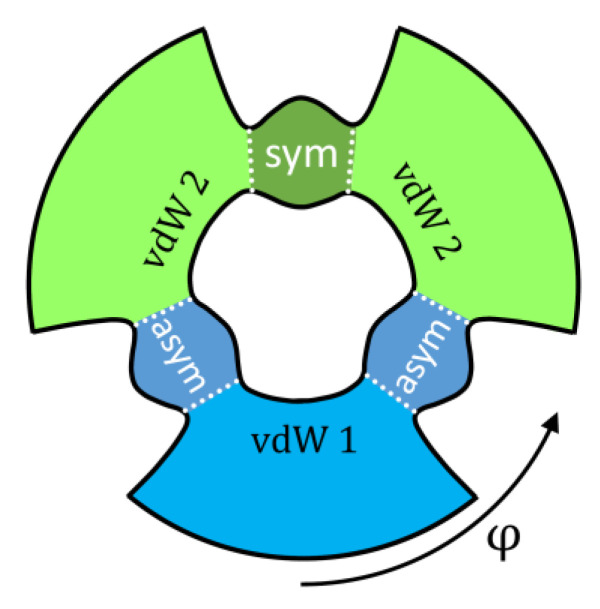
Four areas on the PES of ozone that correspond to the covalent wells with symmetric (green) and asymmetric (blue) ozone molecules, and two types of van der Waals complexes (light blue and light green). Hyper-angle *φ* is used to differentiate between symmetric and asymmetric ozone molecules. Dotted lines are drawn through transition state regions seen as “bottlenecks” in this figure. This diagram can be compared to both frames of Figure 1.

Indeed, the PES of ozone (see Figure 1) contains three deep localized wells that correspond to stable symmetric and asymmetric ozone molecules with covalent bonds. Those wells are connected, through relatively tight transition states (bottlenecks in Figure 1), to the shallow and broad plateaus that host the weakly-bound van der Waals complexes adjacent to the channels [35,36,37]. So, for each resonance state, one can define four probabilities obtained by integrating its wavefunction over four regions of the PES indicated in Figure 4 by different colors: $p_{i}^{\mathrm{sym}}$, $p_{i}^{\mathrm{asym}}$, $p_{i}^{\mathrm{vdw}1}$, $p_{i}^{\mathrm{vdw}2}$. The total covalent probability is $p_{i}^{\mathrm{cov}}=p_{i}^{\mathrm{sym}}+p_{i}^{\mathrm{asym}}$, while the total van der Waals probability is $p_{i}^{\mathrm{vdw}}=\;p_{i}^{\mathrm{vdw}1}+p_{i}^{\mathrm{vdw}2}$. From these data, we can also compute, for each resonance state, the probability of wavefunction in the asymptotic range of the PES: $p_{i}^{\mathrm{free}}=1-\;p_{i}^{\mathrm{vdw}}-p_{i}^{\mathrm{cov}}$, associated with the unbound (scattering) state behavior, similar to free particle states. These data are available for all resonances of ozone in our database.

**Figure 5:**
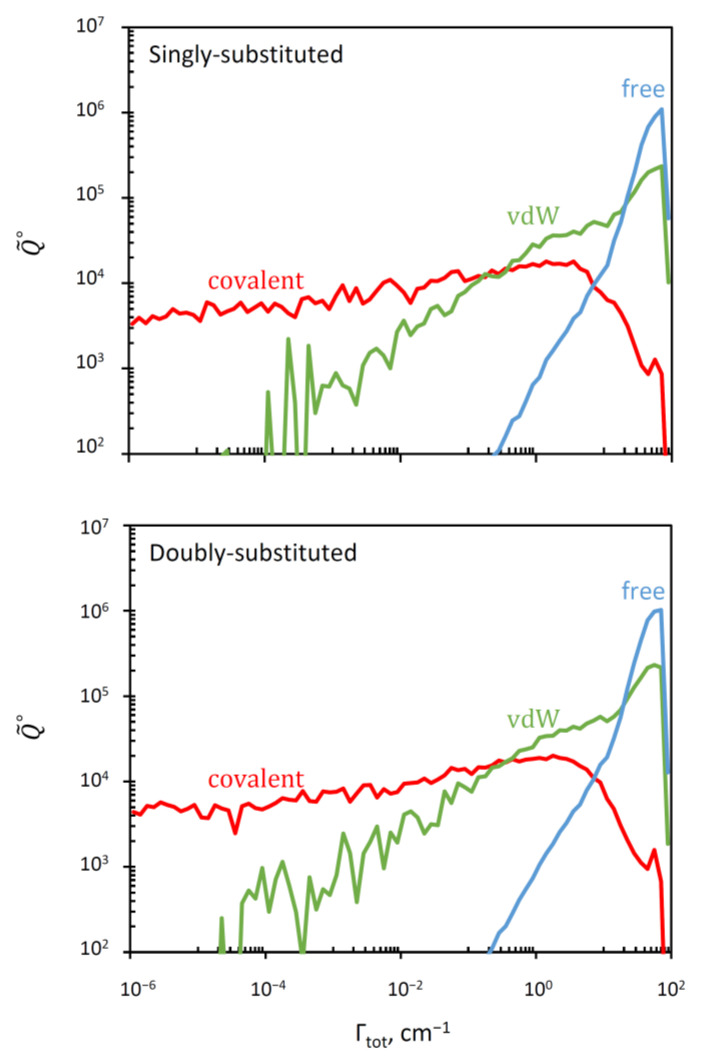
Deconvolution of the overall distribution of resonance widths (given in Figure 3) onto three components that correspond to the covalent well states (**red**), van der Waals plateau states (**green**), and the continuum states in the asymptotic region of the PES (**blue**). The cases of single and double isotopic substitutions are given in the upper and lower frames, respectively.

The results of such deconvolution are presented separately for singly substituted case and for doubly substituted case in the two frames of Figure 5. We see that in the log-log scale used for these histograms, the distribution of resonance width for the covalent well of ozone (red) covers a broad range below Γ ~ 1 cm^−1^, growing roughly linearly, but then drops quickly. At about the same point the number of free-particle states (blue) starts growing rapidly into the range Γ > 1 cm^−1^. The distribution of the van der Waals states (green) overlaps with both distributions, covering the range Γ > 10^−3^ cm^−1^, and also growing roughly linearly in this log-log plot.

Using ${\tilde{Q}}_{}^{\mathrm{cov}}$, ${\tilde{Q}}_{}^{\mathrm{vdw}}$ and ${\tilde{Q}}_{}^{\mathrm{free}}$ it is straightforward to compute the *average* resonance width  Γ ˜ in each group of states (see Supplementary Materials), and the corresponding average lifetime, $\tilde{\tau }\;=\hbar /\;\Gamma \;\text{˜}$. These data are presented in Table 2. They indicate that in ozone the resonances localized in the covalent well are much narrower (more stable) than those distributed over the van der Waals plateau. Indeed, the covalent states are trapped behind the transition state point and therefore they must decay slower (and are populated slower too). In contrast, the wave functions of the van der Waals states are located outside of the transition state, very close to the dissociation channel (see Figure 4). Therefore, they are less stable, they decay (and are populated) much faster.

### 3.4. Resonance Widths in Symmetric and Asymmetric Ozone Molecules

**Figure 6:**
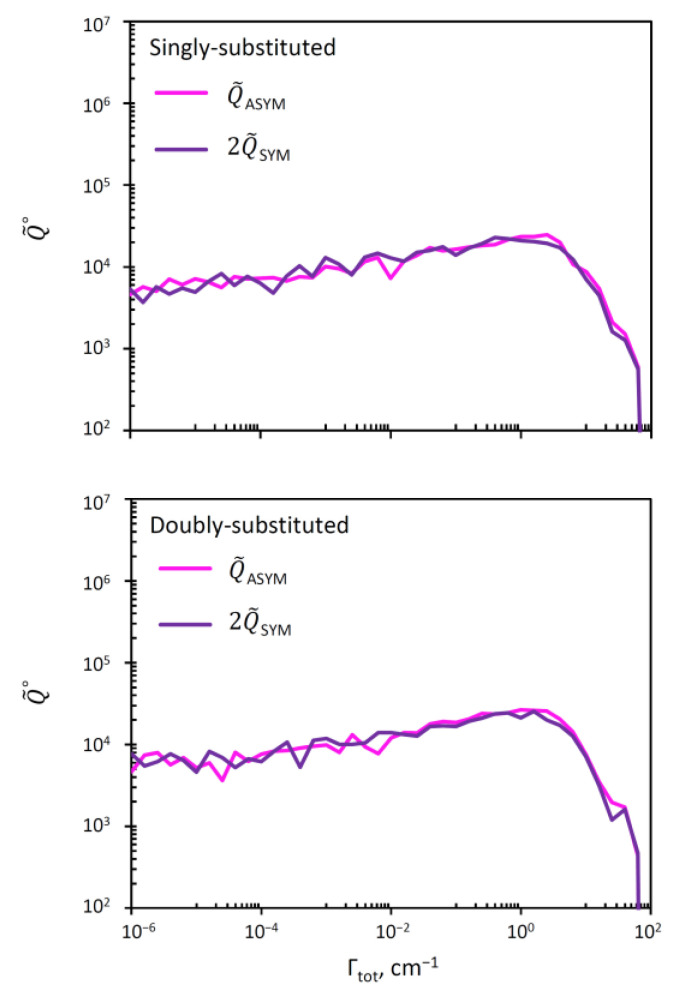
Distribution of resonance widths for the covalent well states in asymmetric (**pink**) and symmetric (**violet**) ozone molecules. Upper and lower frames correspond to single and double isotopic substitutions. To simplify the comparison, we plotted ${\tilde{Q}}_{\mathrm{asym}}^{\circ }$ and $2{\tilde{Q}}_{\mathrm{sym}}^{\circ }$ in order to offset the effect of symmetry.

It was argued by several authors [10,11,38,39] that the lifetimes of metastable states in symmetric and asymmetric ozone molecules may be quite different, and this may explain the symmetry-driven *η*-effect. Lifetimes are inversely proportional to resonance widths, so, here, in order to check this hypothesis, will obtain the distributions of resonance widths in symmetric and asymmetric ozone molecules. For this, we can readily split the overall covalent probabilities between the wells that hold symmetric and asymmetric ozone molecules, using $p_{i}^{\mathrm{cov}}=p_{i}^{\mathrm{sym}}+p_{i}^{\mathrm{asym}}$, and plotting the distributions for ${\tilde{Q}}_{\mathrm{sym}}^{\circ }$ and ${\tilde{Q}}_{\mathrm{asym}}^{\circ }$, individually. This is done in two frames of Figure 6, for the cases of single and double isotopic substitutions. For convenience of comparison, the data for symmetric ozone molecules were multiplied by 2, in order to offset the effect of symmetry (see above). We checked that without this symmetry factor, the sum of two curves in each frame of Figure 6 gives the corresponding red curve in Figure 5. From Figure 6 we can see that the distributions of resonance width are very similar in symmetric and asymmetric ozone molecules in both singly and doubly substituted cases.

Based on these distributions, the average values of resonance width in symmetric and asymmetric ozone molecules were computed and reported in Table 3. We see that the average values of widths and lifetimes are also very similar in symmetric and asymmetric ozone isotopomers, for both single and double isotopic substitutions. Based on these data, we cannot claim that there is a significant difference in the lifetimes of resonances in symmetric and asymmetric ozone molecules, that could be used to explain the symmetry driven *η*-effect.

## 4. Conclusions

In this paper we worked out theoretical basis for the description of all possible recombination pathways in the ozone forming reaction, without neglecting any process *a priori*, and without decoupling the individual pathways one from another. These pathways become physically distinct when a rare isotope of oxygen is introduced, such as ^18^O, and therefore they represent a sensitive probe of fine details in the ozone forming reaction. Here we considered the cases of single and double substitutions with ^18^O, but the cases of isotope ^17^O can be described in a similar manner.

One should realize that, in each isotopic case, on the global potential energy surface of O_3_ there are two types of physically distinct entrance channels (one with homonuclear and the other with heteronuclear oxygen molecule) and two types of physically distinct product wells (one with symmetric and the other with asymmetric ozone molecules). This 2 × 2 combination leads to the appearance of *four* physically distinct recombination pathways that are coupled. A consistent description of these processes can be achieved only if, for each metastable ozone state, there is a method of partitioning the rate of formation/decay between the two reagent channels, and splitting the rate of stabilization between the two product wells.

Using the database of metastable ozone states (scattering resonances computed earlier) as input parameters, we applied this theory to compute rate coefficients for four isotopically labeled recombination pathways in the cases of single and double substitutions with ^18^O, eight rate coefficients total. Based on these data, the nascent isotope effects were determined and compared with available experimental data.

To the best of our knowledge, this is the first theoretical work in which all four rate coefficients are determined simultaneously, including the insertion processes characterized by very small rates. Namely, the insertion of a rare isotope ^18^O in between the atoms in ^16^O^16^O, and the insertion of ^16^O in between the atoms in ^18^O^18^O, are found to be two orders of magnitude slower compared to the other six recombination rates, in agreement with experimental data of the Mauersberger group. Two possible pathways for the formation of each asymmetric ozone molecule (singly substituted ^16^O^16^O^18^O and doubly substituted ^16^O^18^O^18^O) indicate a pronounced, large isotope effect driven by ΔZPE-difference, which is also in good agreement with available experimental data. The rate coefficient for the formation of each symmetric ozone molecule (singly substituted ^16^O^18^O^16^O and doubly substituted ^18^O^16^O^18^O) is found to be in between of those two for the asymmetric ozone molecule, again, in good agreement with experiment. The total formation rates of asymmetric ozone molecules are found to be somewhat larger than those of symmetric ozone molecules but unfortunately, not nearly as much as in the experiment (*η*-effect).

We also took closer look at the distribution of resonance widths that determine lifetimes of the metastable ozone states. The distribution is several orders of magnitude broad. We found that the metastable states of ozone can be characterized based on localization of the wavefunction in different parts of the PES. Namely, the states with wavefunction localized in the covalent ozone well (those that are expected to participate actively in the ozone formation process) are typically narrow (width ~ 1 cm^−1^) and long lived (~33 ps on average). In addition, there are many metastable states with wavefunctions delocalized over the van der Waals plateau of the PES. Such states are found to be less stable, ~ 1.2 ps on average, and are unlikely to participate in the ozone formation process (since their stabilization into the covalent ozone well is expected to be inefficient). Still, the distribution of widths of such resonances is mapped out here, and it is found to be quite different from the distribution of the localized ozone states. However, very similar distributions of resonance properties are found for *symmetric* and *asymmetric* ozone molecules, which seems to disproof a popular hypothesis that the symmetry driven *η*-effect is caused by the difference of lifetimes of the metastable states in symmetric and asymmetric ozone molecules.

The least rigorous component of present theory is a simplified description of stabilization step, which is the second step of the overall recombination process. Here it was assumed that all resonances are stabilized at the same rate. This was done on purpose in some sense, in order to focus on the properties of all resonances, regardless of their position in the spectrum, to determine what isotope effect may come from the properties of the metastable states alone, rather than from the collisions of the metastable ozone with bath gas. Obviously, a more rigorous description of the stabilization step is desirable and will be pursued in the future work.

## Figures and Tables

**Table 1:** Experimental and theoretical rate coefficients (in the units of 10^−35^ cm^6^/s) for four pathways of ozone formation at 296 K in the cases of single and double isotopic substitution, and the corresponding three isotope effects as defined by Equations (61), (64)–(67) in Section 3.1 and Section 3.2 below.

Rate Coefficients	Experiment [1,2,3,4,5,6,7,8]		Theory, This Work	
	Single	Double	Single	Double
2κ_S_	60	62	60	61
2κ_A_	86	55	73	57
κ_B_	55	90	55	73
κ_I_	0.36	1.74	0.18	1.39
**Isotope Effects**				
*ζ*	1.68	1.77	1.45	1.38
*η*	1.13	1.19	1.02	1.08
ξ	4.8		7.9	

**Table 2:** Average resonance widths and their corresponding lifetimes for the states of three types in the singly and doubly substituted ozone molecules.

Resonance Type	$\tilde{\Gamma }\left({\mathrm{cm}}^{-1}\right)$		$\tilde{\tau }\left(\mathrm{ps}\right)$	
	Single	Double	Single	Double
Covalent well	1.00	1.01	33.4	33.0
Van der Waals plateau	27.9	26.9	1.20	1.24
Asymptotic states (free)	68.9	67.3	0.484	0.496

**Table 3:** Average resonance widths and their corresponding lifetimes for the resonances localized in the covalent well of symmetric and asymmetric ozone molecules for the cases of single and double isotopic substitutions.

Isotopomer of Ozone	$\tilde{\Gamma }\left(cm^{-1}\right)$		$\tilde{\tau }\left(ps\right)$	
	Single	Double	Single	Double
Symmetric	1.22	0.91	27.3	36.7
Asymmetric	1.17	0.94	28.5	35.5

## Data Availability

The data presented in this study are available on request from the corresponding author.

## Supplementary Materials

The following are available online. Section A, Section B and Section C of the Supplementary Material contain all formula for the case of doubly substituted ozone and some other useful equations, such as low- and high-pressure limits of the dynamical partition functions. Section D provides some technical details of our calculations.
