# Supplementary material for: Four Isotope-Labeled Recombination Pathways of Ozone Formation
Source: Molecules. 2021 Feb 27;26(5):1289. doi: 10.3390/molecules26051289 (PMC7956848; doi:10.3390/molecules26051289)
Supplement: Supplementary file 1 [file molecules-26-01289-s001.pdf]

Supplementary Material

for

FOUR ISOTOPE-LABELED RECOMBINATION PATHWAYS OF OZONE FORMATION

by

Dmitri Babikov, Elizaveta Grushnikova, Igor Gayday and Alexander Teplukhin

A. Rate expressions for double isotopic substitution in ozone

In many aspects the theoretical treatment of the doubly substituted isotopologue of ozone is similar to that of the singly substituted isotopologue (developed in in the main text), but there is one critical difference that makes this case important on its own and requires a careful revamp of all relevant equations. Namely, in the doubly substituted case, Channel 1 that hosts the reagents with symmetric homonuclear oxygen molecule ($^{18}O^{18}O$ + ^{16}O) becomes the lower energy channel and has to be taken as a reference of energy for all the metastable reaction intermediates (scattering resonances). This is demonstrated by Figure S1 below and is different from Figure 2 of the main text for the singly substituted case. Due to this difference, the ΔZPE effect in the experiment switches its direction in the doubly substituted case, compared to the singly substituted case. Theoretical treatment of this effect should also be modified appropriately, as follows.

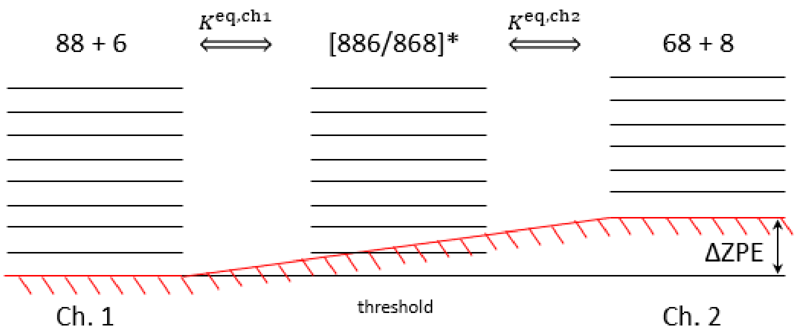

Figure S1. Two distinct channels of ozone formation (left and right) and the metastable ozone states (middle) in the case of double isotopic substitution. Here “6” denotes ^{16}O whereas “8” denotes ^{18}O. Lower energy channel corresponds to the heavier diatomic reagent $^{18}O^{18}O$ with smaller zero-point energy. Upper channel corresponds to the lighter diatomic reagent $^{16}O^{18}O$. Energy difference of the two channels, ΔZPE, is indicated.

In the doubly substituted case the processes of formation, decay and stabilization of scattering resonances (metastable intermediates) of ozone are represented by the following reaction scheme:

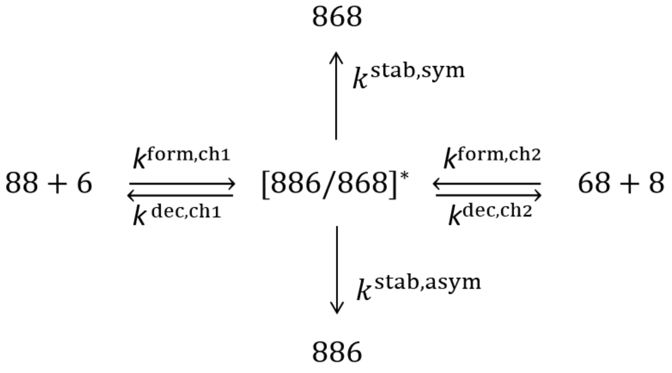

(S1)

Including these processes in the rate of change expression for the concentrations of intermediate species we can write:

$$\frac{d[O_3^*]_i}{dt} = k_i^{\text{form,ch1}}[88][6] + k_i^{\text{form,ch2}}[68][8]$$
$$- k_i^{\text{dec,ch1}}[O_3^*]_i - k_i^{\text{dec,ch2}}[O_3^*]_i - k_i^{\text{stab,sym}}[M][O_3^*]_i - k_i^{\text{stab,asym}}[M][O_3^*]_i \quad \text{(S2)}$$

In the steady state conditions, applicable to ozone formation reaction, we have:

$$0 = k_i^{\text{form,ch1}}[88][6] + k_i^{\text{form,ch2}}[68][8] - k_i^{\text{dec}}[\text{O}_3^*]_i - k_i^{\text{stab}}[\text{M}][\text{O}_3^*]_i \tag{S3}$$

$$[\text{O}_3^*]_i = \frac{k_i^{\text{form,ch1}}[88][6] + k_i^{\text{form,ch2}}[68][8]}{k_i^{\text{dec}} + k_i^{\text{stab}}[\text{M}]} \tag{S4}$$

As before, the rate coefficients for formation of resonances through two channels are converted into the corresponding rate coefficients of decay using two equilibrium constants, but here, in the case of double isotopic substitutions, the expressions are different:

$$K_i^{\text{eq,ch1}} = \frac{(2J+1)\, e^{-E_i/kT}}{Q_{\text{ch1}}} \tag{S5}$$

$$K_i^{\text{eq,ch2}} = \frac{(2J+1)\, e^{-E_i/kT}}{Q_{\text{ch2}} e^{-\Delta\text{ZPE}/kT}} \tag{S6}$$

Namely, here the $e^{-\Delta\text{ZPE}/kT}$ factor shows up in Channel 2 (in contrast with the singly substituted case, where it appears in Channel 1). The value of ΔZPE itself in the doubly substituted case is also slightly different than in the singly substituted case. Here we have:

$$\Delta\text{ZPE} = \text{ZPE}(68) - \text{ZPE}(88) = 22.94\ \text{cm}^{-1}$$

Using these formulae, we obtain the following expressions for two product-specific rates of recombination process:

$$\begin{aligned} R_{\text{sym}} = {} & [\text{M}][68][8]\frac{k^{\text{stab}}}{Q_{\text{ch2}}}\sum_i (2J+1) p_i^{\text{sym}} \frac{\frac{\Gamma_i^{\text{ch2}}}{\hbar}}{\frac{\Gamma_i^{\text{tot}}}{\hbar} + k_i^{\text{stab}}[\text{M}]} e^{-\frac{E_i - \Delta\text{ZPE}}{kT}} \\ & + [\text{M}][88][6]\frac{k^{\text{stab}}}{Q_{\text{ch1}}}\sum_i (2J+1) p_i^{\text{sym}} \frac{\frac{\Gamma_i^{\text{ch1}}}{\hbar}}{\frac{\Gamma_i^{\text{tot}}}{\hbar} + k_i^{\text{stab}}[\text{M}]} e^{-\frac{E_i}{kT}} \end{aligned} \tag{S7}$$

$$R_{\mathrm{asym}} = [\mathrm{M}][68][8]\,\frac{k^{\mathrm{stab}}}{Q_{\mathrm{ch2}}}\sum_i (2J+1)p_i^{\mathrm{asym}}\,\frac{\frac{\Gamma_i^{\mathrm{ch2}}}{\hbar}}{\frac{\Gamma_i^{\mathrm{tot}}}{\hbar}+k_i^{\mathrm{stab}}[\mathrm{M}]}\,e^{-\frac{E_i-\Delta\mathrm{ZPE}}{kT}}$$
$$+ [\mathrm{M}][88][6]\,\frac{k^{\mathrm{stab}}}{Q_{\mathrm{ch1}}}\sum_i (2J+1)p_i^{\mathrm{asym}}\,\frac{\frac{\Gamma_i^{\mathrm{ch1}}}{\hbar}}{\frac{\Gamma_i^{\mathrm{tot}}}{\hbar}+k_i^{\mathrm{stab}}[\mathrm{M}]}\,e^{-\frac{E_i}{kT}} \tag{S8}$$

The following four pathway-specific dynamical partition functions can be introduced to simplify these expressions:

$$\tilde{Q}_{\mathrm{A}} \equiv \sum_i (2J+1)\,w_i\frac{\Gamma_i^{\mathrm{ch2}}}{\Gamma_i^{\mathrm{tot}}}\,p_i^{\mathrm{asym}}\,e^{-\frac{E_i-\Delta\mathrm{ZPE}}{kT}} \tag{S9}$$

$$\tilde{Q}_{\mathrm{B}} \equiv \sum_i (2J+1)\,w_i\frac{\Gamma_i^{\mathrm{ch1}}}{\Gamma_i^{\mathrm{tot}}}\,p_i^{\mathrm{asym}}e^{-\frac{E_i}{kT}} \tag{S10}$$

$$\tilde{Q}_{\mathrm{S}} \equiv \sum_i (2J+1)\,w_i\frac{\Gamma_i^{\mathrm{ch2}}}{\Gamma_i^{\mathrm{tot}}}\,p_i^{\mathrm{sym}}e^{-\frac{E_i-\Delta\mathrm{ZPE}}{kT}} \tag{S11}$$

$$\tilde{Q}_{\mathrm{I}} \equiv \sum_i (2J+1)\,w_i\frac{\Gamma_i^{\mathrm{ch1}}}{\Gamma_i^{\mathrm{tot}}}\,p_i^{\mathrm{sym}}e^{-\frac{E_i}{kT}} \tag{S12}$$

Note that here the ΔZPE shift appears in the expressions for $\tilde{Q}_{\mathrm{A}}$ and $\tilde{Q}_{\mathrm{S}}$, while in the case of single isotopic substitution it appeared in $\tilde{Q}_{\mathrm{B}}$ and $\tilde{Q}_{\mathrm{I}}$ (see Equations 41-44 of the main text).

The insertion pathway is shown schematically in Fig. S2 below, for the case of double isotopic substitution. The case of single isotopic substitution is similar.

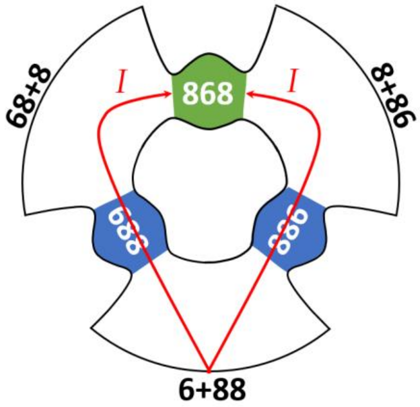

Figure S2. Schematic of the global PES of ozone that possesses a three-fold symmetry with respect to the entrance channels and the product wells. Reagents are indicated by black numbers, the product ozone molecules by white numbers, for the case of double isotopic substitution. The insertion pathway is shown schematically by red arrows.

The isotope exchange process, and the corresponding equilibrium constant, in the case of double substitution are:

$$88 + 6 \rightleftharpoons 68 + 8 \tag{S13}$$

$$K^{\text{ex}} = \frac{[68][8]}{[88][6]} = \frac{Q_{\text{ch2}} e^{-\Delta\text{ZPE}/kT}}{Q_{\text{ch1}}} \tag{S14}$$

where the factor $e^{-\Delta\text{ZPE}/kT}$ is used in Channel 2. The forward direction of the isotope exchange here is defined as from Channel 1 to Channel 2, opposite to the singly substituted case. The value of this equilibrium constant is expected to be on the order of 2 due to symmetry of the homonuclear diatomic reagent in the Channel 1, in which every other rotational state is forbidden by symmetry. Using this expression, we can rewrite formula for the rates in the way where all pathways are referenced relative to the lowest energy channel, as follows:

$$R_{\text{sym}} = R_{\text{S}} + R_{\text{I}} = \kappa_{\text{S}}[\text{M}][68][8] + \kappa_{\text{I}}[\text{M}][88][6]$$
$$= (\kappa_{\text{S}} \frac{[68][8]}{[88][6]} + \kappa_{\text{I}})[88][6][\text{M}] = \kappa_{\text{sym}}[88][6][\text{M}] \tag{S15}$$

$$R_{\text{asym}} = R_{\text{A}} + R_{\text{B}} = \kappa_{\text{A}}[\text{M}][68][8] + \kappa_{\text{B}}[\text{M}][88][6]$$
$$= (\kappa_{\text{A}}\frac{[68][8]}{[88][6]} + \kappa_{\text{B}})[88][6][\text{M}] = \kappa_{\text{asym}}[88][6][\text{M}] \quad \text{(S16)}$$

A relationship between the pathway-specific rate coefficients and the product-specific rate coefficients then looks as follows:

$$\kappa_{\text{sym}} = \kappa_{\text{S}}K^{\text{ex}} + \kappa_{\text{I}} = k^{\text{stab}}\frac{\tilde{Q}_{\text{sym}}}{Q_{\text{ch1}}} \quad \text{(S17)}$$

$$\kappa_{\text{asym}} = \kappa_{\text{A}}K^{\text{ex}} + \kappa_{\text{B}} = k^{\text{stab}}\frac{\tilde{Q}_{\text{asym}}}{Q_{\text{ch1}}} \quad \text{(S18)}$$

Note that here, in the doubly substituted case, the K^{ex} sticks to the rate coefficients of pathways A and S that originate in the higher energy channel, the Channel 2. Interestingly, the equations for product-specific dynamical partition functions $\tilde{Q}_{\text{sym}}$ and $\tilde{Q}_{\text{asym}}$ in the doubly-substituted case appear to be entirely identical to those derived for the singly-substituted case, simply because these formula do not contain any $e^{-\Delta\text{ZPE}/kT}$ factors (they analytically cancel, as it was discussed in Sec. II of the main text). Therefore, they will not be repeated here.

B. Rate expressions for channel-specific processes

Since $R_{\text{tot}} = R_{\text{A}} + R_{\text{B}} + R_{\text{S}} + R_{\text{I}}$, we can regroup the four contributions into two rates that correspond to two entrance channels:

$$R_{\text{ch2}} = R_{\text{A}} + R_{\text{S}} \quad \text{(S19)}$$

$$R_{\text{ch1}} = R_{\text{B}} + R_{\text{I}} \quad \text{(S20)}$$

First consider the case of single isotopic substitution. Using the definitions of Eqs. (36-39, 45-48) of the main text, one immediately obtains expressions for the corresponding rate coefficients:

$$\kappa_{\mathrm{ch1}} = \kappa_{\mathrm{B}} + \kappa_{\mathrm{I}} = k^{\mathrm{stab}} \frac{\tilde{Q}_{\mathrm{B}} + \tilde{Q}_{\mathrm{I}}}{Q_{\mathrm{ch1}}} \tag{S21}$$

$$\kappa_{\mathrm{ch2}} = \kappa_{\mathrm{A}} + \kappa_{\mathrm{S}} = k^{\mathrm{stab}} \frac{\tilde{Q}_{\mathrm{A}} + \tilde{Q}_{\mathrm{S}}}{Q_{\mathrm{ch2}}} \tag{S22}$$

Two dynamical partition functions for these processes can be defined as a simple sum:

$$\tilde{Q}_{\mathrm{ch1}} \equiv \tilde{Q}_{\mathrm{B}} + \tilde{Q}_{\mathrm{I}} \tag{S23}$$

$$\tilde{Q}_{\mathrm{ch2}} \equiv \tilde{Q}_{\mathrm{A}} + \tilde{Q}_{\mathrm{S}} \tag{S24}$$

Using Equation 41-44 of the main text we obtain the following expressions (through properties of the individual resonances):

$$\tilde{Q}_{\mathrm{ch1}} \equiv \sum_{i} (2J+1)\, w_i \frac{\Gamma_i^{\mathrm{ch1}}}{\Gamma_i^{\mathrm{tot}}}\, p_i^{\mathrm{tot}}\, e^{-\frac{E_i - \Delta\mathrm{ZPE}}{kT}} \tag{S25}$$

$$\tilde{Q}_{\mathrm{ch2}} \equiv \sum_{i} (2J+1)\, w_i \frac{\Gamma_i^{\mathrm{ch2}}}{\Gamma_i^{\mathrm{tot}}}\, p_i^{\mathrm{tot}}\, e^{-\frac{E_i}{kT}} \tag{S26}$$

This result is also quite interesting. These expressions indicate that the splitting of p_i^{tot} onto p_i^{sym} and p_i^{asym} has no influence on $\tilde{Q}_{\mathrm{ch1}}$ vs. $\tilde{Q}_{\mathrm{ch2}}$ since the same overall stabilization probability enters both expressions. In this case the difference between $\tilde{Q}_{\mathrm{ch1}}$ and $\tilde{Q}_{\mathrm{ch2}}$ comes entirely from splitting of Γ_i^{tot} onto Γ_i^{ch1} and Γ_i^{ch2}, for individual resonances. In this procedure, Γ_i^{ch1} comes with the ΔZPE shift of energy reference, as emphasized above.

In the doubly substituted case:

$$\tilde{Q}_{\mathrm{ch1}} \equiv \tilde{Q}_{\mathrm{B}} + \tilde{Q}_{\mathrm{I}} = \sum_{i} (2J+1)\, w_i \frac{\Gamma_i^{\mathrm{ch1}}}{\Gamma_i^{\mathrm{tot}}}\, p_i^{\mathrm{tot}}\, e^{-\frac{E_i}{kT}} \tag{S27}$$

$$\tilde{Q}_{\mathrm{ch2}} \equiv \tilde{Q}_{\mathrm{A}} + \tilde{Q}_{\mathrm{S}} = \sum_{i} (2J+1)\, w_i \frac{\Gamma_i^{\mathrm{ch2}}}{\Gamma_i^{\mathrm{tot}}}\, p_i^{\mathrm{tot}}\, e^{-\frac{E_i - \Delta\mathrm{ZPE}}{kT}} \tag{S28}$$

Again, the difference from the singly substituted case is that here the $e^{-\Delta\mathrm{ZPE}/kT}$ factor comes with the upper Channel 2.

C. Rate expressions for low-pressure and high-pressure limits

Low-pressure limit is relevant to the atmospheric chemistry, to some laboratory studies, but is also important methodologically. In the limit of zero pressure of bath gas, $[\mathrm{M}] = 0$, the weights of all resonances achieve their maximum value $w_i = 1$, which simplifies the equations as follows (in the case of single-substitution):

$$\tilde{Q}^{\circ}_{\mathrm{tot}} = \sum_i (2J+1)\, p_i^{\mathrm{tot}}\, e^{-\frac{E_i}{kT}} \tag{S29}$$

$$\tilde{Q}^{\circ}_{\mathrm{sym}} = \sum_i (2J+1)\, p_i^{\mathrm{sym}}\, e^{-\frac{E_i}{kT}} \tag{S30}$$

$$\tilde{Q}^{\circ}_{\mathrm{asym}} = \sum_i (2J+1)\, p_i^{\mathrm{asym}}\, e^{-\frac{E_i}{kT}} \tag{S31}$$

$$\tilde{Q}^{\circ}_{\mathrm{ch1}} = \sum_i (2J+1)\, \frac{\Gamma_i^{\mathrm{ch1}}}{\Gamma_i^{\mathrm{tot}}}\, p_i^{\mathrm{tot}}\, e^{-\frac{E_i - \Delta\mathrm{ZPE}}{kT}} \tag{S32}$$

$$\tilde{Q}^{\circ}_{\mathrm{ch2}} = \sum_i (2J+1)\, \frac{\Gamma_i^{\mathrm{ch2}}}{\Gamma_i^{\mathrm{tot}}}\, p_i^{\mathrm{tot}}\, e^{-\frac{E_i}{kT}} \tag{S33}$$

$$\tilde{Q}^{\circ}_{\mathrm{A}} = \sum_i (2J+1)\, \frac{\Gamma_i^{\mathrm{ch2}}}{\Gamma_i^{\mathrm{tot}}}\, p_i^{\mathrm{asym}}\, e^{-\frac{E_i}{kT}} \tag{S34}$$

$$\tilde{Q}^{\circ}_{\mathrm{B}} = \sum_i (2J+1)\, \frac{\Gamma_i^{\mathrm{ch1}}}{\Gamma_i^{\mathrm{tot}}}\, p_i^{\mathrm{asym}} e^{-\frac{E_i - \Delta\mathrm{ZPE}}{kT}} \tag{S35}$$

$$\tilde{Q}_{\mathrm{S}}^{\circ} = \sum_i (2J+1) \frac{\Gamma_i^{\mathrm{ch2}}}{\Gamma_i^{\mathrm{tot}}} p_i^{\mathrm{sym}} e^{-\frac{E_i}{kT}} \tag{S36}$$

$$\tilde{Q}_{\mathrm{I}}^{\circ} = \sum_i (2J+1) \frac{\Gamma_i^{\mathrm{ch1}}}{\Gamma_i^{\mathrm{tot}}} p_i^{\mathrm{sym}} e^{-\frac{E_i - \Delta\mathrm{ZPE}}{kT}} \tag{S37}$$

An important point to emphasize is that $\tilde{Q}_{\mathrm{tot}}^{\circ}$, $\tilde{Q}_{\mathrm{sym}}^{\circ}$ and $\tilde{Q}_{\mathrm{asym}}^{\circ}$ do not include the widths of resonances, at all. Therefore, in the limit of zero-pressure any isotope effects in symmetric vs. asymmetric ozone molecules driven by resonance width (lifetimes of the metastable states) are expected to disappear. The expressions for $\tilde{Q}_{\mathrm{ch1}}^{\circ}$, $\tilde{Q}_{\mathrm{ch2}}^{\circ}$, $\tilde{Q}_{\mathrm{A}}^{\circ}$, $\tilde{Q}_{\mathrm{B}}^{\circ}$, $\tilde{Q}_{\mathrm{S}}^{\circ}$ and $\tilde{Q}_{\mathrm{I}}^{\circ}$ still contain resonance widths, but only in the relative ratios $\Gamma_i^{\mathrm{ch1}}/\Gamma_i^{\mathrm{tot}}$ and $\Gamma_i^{\mathrm{ch2}}/\Gamma_i^{\mathrm{tot}}$. Therefore, the absolute values of resonance widths do not really matter. What remains important is a partition (splitting) of Γ_i^{tot} between the two reaction channels.

In the high-pressure limit $[\mathrm{M}] \to \infty$ the resonance weight decreases as a function of pressure and increases as a function of width:

$$w_i \approx \frac{\frac{\Gamma_i^{\mathrm{tot}}}{\hbar}}{k_i^{\mathrm{stab}}[\mathrm{M}]} \tag{S38}$$

This leads to the following expressions:

$$\tilde{Q}_{\mathrm{tot}}^{\infty} = \sum_i (2J+1) \frac{\Gamma_i^{\mathrm{tot}}}{\hbar k^{\mathrm{stab}}[\mathrm{M}]} e^{-\frac{E_i}{kT}} \tag{S39}$$

$$\tilde{Q}_{\mathrm{sym}}^{\infty} = \sum_i (2J+1) \frac{\Gamma_i^{\mathrm{tot}}}{\hbar k^{\mathrm{stab}}[\mathrm{M}]} \frac{p_i^{\mathrm{sym}}}{p_i^{\mathrm{tot}}} e^{-\frac{E_i}{kT}} \tag{S40}$$

$$\tilde{Q}_{\mathrm{asym}}^{\infty} = \sum_i (2J+1) \frac{\Gamma_i^{\mathrm{tot}}}{\hbar k^{\mathrm{stab}}[\mathrm{M}]} \frac{p_i^{\mathrm{asym}}}{p_i^{\mathrm{tot}}} e^{-\frac{E_i}{kT}} \tag{S41}$$

$$\tilde{Q}^{\infty}_{\mathrm{ch1}} = \sum_i (2J+1)\ \frac{\Gamma_i^{\mathrm{ch1}}}{\hbar k^{\mathrm{stab}}[\mathrm{M}]}\ e^{-\frac{E_i-\Delta\mathrm{ZPE}}{kT}} \tag{S42}$$

$$\tilde{Q}^{\infty}_{\mathrm{ch2}} = \sum_i (2J+1)\ \frac{\Gamma_i^{\mathrm{ch2}}}{\hbar k^{\mathrm{stab}}[\mathrm{M}]}\ e^{-\frac{E_i}{kT}} \tag{S43}$$

$$\tilde{Q}^{\infty}_{\mathrm{A}} = \sum_i (2J+1) \frac{\Gamma_i^{\mathrm{ch2}}}{\hbar k^{\mathrm{stab}}[\mathrm{M}]} \frac{p_i^{\mathrm{asym}}}{p_i^{\mathrm{tot}}} e^{-\frac{E_i}{kT}} \tag{S44}$$

$$\tilde{Q}^{\infty}_{\mathrm{B}} = \sum_i (2J+1) \frac{\Gamma_i^{\mathrm{ch1}}}{\hbar k^{\mathrm{stab}}[\mathrm{M}]} \frac{p_i^{\mathrm{asym}}}{p_i^{\mathrm{tot}}} e^{-\frac{E_i-\Delta\mathrm{ZPE}}{kT}} \tag{S45}$$

$$\tilde{Q}^{\infty}_{\mathrm{S}} = \sum_i (2J+1) \frac{\Gamma_i^{\mathrm{ch2}}}{\hbar k^{\mathrm{stab}}[\mathrm{M}]} \frac{p_i^{\mathrm{sym}}}{p_i^{\mathrm{tot}}} e^{-\frac{E_i}{kT}} \tag{S46}$$

$$\tilde{Q}^{\infty}_{\mathrm{I}} = \sum_i (2J+1) \frac{\Gamma_i^{\mathrm{ch1}}}{\hbar k^{\mathrm{stab}}[\mathrm{M}]} \frac{p_i^{\mathrm{sym}}}{p_i^{\mathrm{tot}}} e^{-\frac{E_i-\Delta\mathrm{ZPE}}{kT}} \tag{S47}$$

We see that in this limiting case $\tilde{Q}^{\infty}_{\mathrm{tot}}$, $\tilde{Q}^{\infty}_{\mathrm{sym}}$ and $\tilde{Q}^{\infty}_{\mathrm{asym}}$ contain Γ_i^{tot}, while $\tilde{Q}^{\infty}_{\mathrm{ch1}}$, $\tilde{Q}^{\infty}_{\mathrm{ch2}}$, $\tilde{Q}^{\infty}_{\mathrm{A}}$, $\tilde{Q}^{\infty}_{\mathrm{B}}$, $\tilde{Q}^{\infty}_{\mathrm{S}}$ and $\tilde{Q}^{\infty}_{\mathrm{I}}$ contain either Γ_i^{ch1} or Γ_i^{ch2}. But, neither of these come as a ratio, so, the absolute values of resonance widths are important in the high-pressure regime. In contrast, the absolute values of stabilization probabilities become unimportant. They either completely cancel, as in $\tilde{Q}^{\infty}_{\mathrm{tot}}$, $\tilde{Q}^{\infty}_{\mathrm{ch1}}$ and $\tilde{Q}^{\infty}_{\mathrm{ch2}}$, or come to play but only as relative ratios $p_i^{\mathrm{sym}}/p_i^{\mathrm{tot}}$ and $p_i^{\mathrm{sym}}/p_i^{\mathrm{tot}}$ in expressions for $\tilde{Q}^{\infty}_{\mathrm{sym}}$, $\tilde{Q}^{\infty}_{\mathrm{asym}}$, $\tilde{Q}^{\infty}_{\mathrm{A}}$, $\tilde{Q}^{\infty}_{\mathrm{B}}$, $\tilde{Q}^{\infty}_{\mathrm{S}}$ and $\tilde{Q}^{\infty}_{\mathrm{I}}$.

For completeness, we list all these formula for the low- and high-pressure in the doubly-substituted case:

$$\tilde{Q}^{\circ}_{\mathrm{tot}} = \sum_i (2J+1)\ p_i^{\mathrm{tot}}\ e^{-\frac{E_i}{kT}} \tag{S48}$$

$$\tilde{Q}^{\circ}_{\mathrm{sym}} = \sum_i (2J+1)\ p_i^{\mathrm{sym}}\ e^{-\frac{E_i}{kT}} \tag{S49}$$

$$\tilde{Q}^{\circ}_{\mathrm{asym}} = \sum_i (2J+1)\, p_i^{\mathrm{asym}}\, e^{-\frac{E_i}{kT}} \tag{S50}$$

$$\tilde{Q}^{\circ}_{\mathrm{ch1}} = \sum_i (2J+1)\, \frac{\Gamma_i^{\mathrm{ch1}}}{\Gamma_i^{\mathrm{tot}}}\, p_i^{\mathrm{tot}}\, e^{-\frac{E_i}{kT}} \tag{S51}$$

$$\tilde{Q}^{\circ}_{\mathrm{ch2}} = \sum_i (2J+1)\, \frac{\Gamma_i^{\mathrm{ch2}}}{\Gamma_i^{\mathrm{tot}}}\, p_i^{\mathrm{tot}}\, e^{-\frac{E_i - \Delta\mathrm{ZPE}}{kT}} \tag{S52}$$

$$\tilde{Q}^{\circ}_{\mathrm{A}} = \sum_i (2J+1)\, \frac{\Gamma_i^{\mathrm{ch2}}}{\Gamma_i^{\mathrm{tot}}}\, p_i^{\mathrm{asym}}\, e^{-\frac{E_i - \Delta\mathrm{ZPE}}{kT}} \tag{S53}$$

$$\tilde{Q}^{\circ}_{\mathrm{B}} = \sum_i (2J+1)\, \frac{\Gamma_i^{\mathrm{ch1}}}{\Gamma_i^{\mathrm{tot}}}\, p_i^{\mathrm{asym}}\, e^{-\frac{E_i}{kT}} \tag{S54}$$

$$\tilde{Q}^{\circ}_{\mathrm{S}} = \sum_i (2J+1)\, \frac{\Gamma_i^{\mathrm{ch2}}}{\Gamma_i^{\mathrm{tot}}}\, p_i^{\mathrm{sym}}\, e^{-\frac{E_i - \Delta\mathrm{ZPE}}{kT}} \tag{S55}$$

$$\tilde{Q}^{\circ}_{\mathrm{I}} = \sum_i (2J+1)\, \frac{\Gamma_i^{\mathrm{ch1}}}{\Gamma_i^{\mathrm{tot}}}\, p_i^{\mathrm{sym}}\, e^{-\frac{E_i}{kT}} \tag{S56}$$

$$\tilde{Q}^{\infty}_{\mathrm{tot}} = \sum_i (2J+1)\, \frac{\Gamma_i^{\mathrm{tot}}}{\hbar k^{\mathrm{stab}}[\mathrm{M}]}\, e^{-\frac{E_i}{kT}} \tag{S57}$$

$$\tilde{Q}^{\infty}_{\mathrm{sym}} = \sum_i (2J+1)\, \frac{\Gamma_i^{\mathrm{tot}}}{\hbar k^{\mathrm{stab}}[\mathrm{M}]}\, \frac{p_i^{\mathrm{sym}}}{p_i^{\mathrm{tot}}}\, e^{-\frac{E_i}{kT}} \tag{S58}$$

$$\tilde{Q}^{\infty}_{\mathrm{asym}} = \sum_i (2J+1)\, \frac{\Gamma_i^{\mathrm{tot}}}{\hbar k^{\mathrm{stab}}[\mathrm{M}]}\, \frac{p_i^{\mathrm{asym}}}{p_i^{\mathrm{tot}}}\, e^{-\frac{E_i}{kT}} \tag{S59}$$

$$\tilde{Q}^{\infty}_{\mathrm{ch1}} = \sum_i (2J+1)\, \frac{\Gamma_i^{\mathrm{ch1}}}{\hbar k^{\mathrm{stab}}[\mathrm{M}]}\, e^{-\frac{E_i}{kT}} \tag{S60}$$

$$\tilde{Q}^{\infty}_{\mathrm{ch2}} = \sum_i (2J+1)\, \frac{\Gamma_i^{\mathrm{ch2}}}{\hbar k^{\mathrm{stab}}[\mathrm{M}]}\, e^{-\frac{E_i - \Delta\mathrm{ZPE}}{kT}} \tag{S61}$$

$$\tilde{Q}_{\mathrm{A}}^{\infty} = \sum_i (2J+1) \frac{\Gamma_i^{\mathrm{ch2}}}{\hbar k^{\mathrm{stab}}[\mathrm{M}]} \frac{p_i^{\mathrm{asym}}}{p_i^{\mathrm{tot}}} e^{-\frac{E_i - \Delta \mathrm{ZPE}}{kT}} \tag{S62}$$

$$\tilde{Q}_{\mathrm{B}}^{\infty} = \sum_i (2J+1) \frac{\Gamma_i^{\mathrm{ch1}}}{\hbar k^{\mathrm{stab}}[\mathrm{M}]} \frac{p_i^{\mathrm{asym}}}{p_i^{\mathrm{tot}}} e^{-\frac{E_i}{kT}} \tag{S63}$$

$$\tilde{Q}_{\mathrm{S}}^{\infty} = \sum_i (2J+1) \frac{\Gamma_i^{\mathrm{ch2}}}{\hbar k^{\mathrm{stab}}[\mathrm{M}]} \frac{p_i^{\mathrm{sym}}}{p_i^{\mathrm{tot}}} e^{-\frac{E_i - \Delta \mathrm{ZPE}}{kT}} \tag{S64}$$

$$\tilde{Q}_{\mathrm{I}}^{\infty} = \sum_i (2J+1) \frac{\Gamma_i^{\mathrm{ch1}}}{\hbar k^{\mathrm{stab}}[\mathrm{M}]} \frac{p_i^{\mathrm{sym}}}{p_i^{\mathrm{tot}}} e^{-\frac{E_i}{kT}} \tag{S65}$$

D. Technical details of calculations

The following changes were made in this work, relative to the earlier work of Teplukhin et al.[14]:

a) The value of temperature was changed from 296 K used by Teplukhin to $T = 293$ K used here, since the experiments of Mauersberger were conducted at this temperature. Note that temperature enters the factor $e^{-\Delta\mathrm{ZPE}/kT}$ in all the equations derived above, the standard expressions for electronic, translational, and rotational partition functions of reagents, the ideal gas equation to determine concentration [M] at given pressure, and the formula for RMS speed of molecules to express k^{stab} through σ_{stab}. Since T-dependences are relatively smooth (the reactions are only slightly exoergic and endoergic), this correction resulted in a relatively small change.

b) In order to treat the upper and lower channels of the recombination reaction equally, the contributions of resonances below dissociation thresholds were neglected. Such contributions are typically small and originate from the numerical errors of calculations, that predict some non-zero widths (decay rates) to the states slightly below dissociation threshold, which is unphysical. In the previous work all bound states below the lower

dissociation threshold ($E_i < 0$) were correctly neglected, but the resonances within ΔZPE energy range were allowed to decay onto both Channels 1 and 2. This is unphysical since the upper channel (Channel 1 in the singly substituted case and Channel 2 in the doubly substituted case) is still closed at energies $0 < E_i < \Delta\text{ZPE}$. Therefore, it makes sense to neglect the decay of resonances through the upper channel for $E_i < \Delta\text{ZPE}$. Moreover, the energy of dissociation thresholds grows with the quantum number K (body-fixed Z-projection of J), since for the symmetric-top rotor case this is a good quantum number that correlates with rotational quantum number j of the diatomic reagent (66, 68 or 88). For ozone molecule with the quantum number K only the states of a diatomic reagent with $j \geq K$ are permitted asymptotically, making the threshold energies rotation-dependent: $E_i > K(K+1)B_{\text{lower}}$ for the lower channel and $E_i > \Delta\text{ZPE} + K(K+1)B_{\text{upper}}$ for the upper channels. Note that the values of diatomic rotational constant B are different for the two channels and are different in the singly and doubly substituted cases too. The values of vibrational ΔZPE are also different in the singly and doubly substituted cases. And the upper and lower channels switch in the singly and doubly substituted cases. Since the overall picture is this complex and it is hard to estimate the overall effect of all these details, we decided, in the updated version of theory, to take into account all these factors, in order to avoid any bias. This modification also resulted in a relatively small change.

c) Finally, a bug was discovered in the code of Teplukhin, where the reduced mass of $^{16}O^{16}O$ was used erroneously for the pathway B in the doubly substituted case (should be $^{18}O^{18}O$). This was corrected in the updated version of the code, and resulted in a significant change of κ_{B} and K^{ex}, but no change in the isotope effects where these two moieties enter together and the effect of the diatomic mass cancels.

The characteristic values of resonance widths for three kinds of states were computed as average using the following formula:

$$\tilde{\Gamma}_{\mathrm{cov}} = \frac{\sum_i \tilde{Q}_i^{\mathrm{cov}}\, \Gamma_i}{\sum_i \tilde{Q}_i^{\mathrm{cov}}}$$

$$\tilde{\Gamma}_{\mathrm{vdw}} = \frac{\sum_i \tilde{Q}_i^{\mathrm{vdw}}\, \Gamma_i}{\sum_i \tilde{Q}_i^{\mathrm{vdw}}}$$

$$\tilde{\Gamma}_{\mathrm{free}} = \frac{\sum_i \tilde{Q}_i^{\mathrm{free}}\, \Gamma_i}{\sum_i \tilde{Q}_i^{\mathrm{free}}}$$

where $\tilde{Q}_i^{xxx} = (2J+1)\, e^{-\frac{E_i}{kT}}\, w_i\, p_i^{xxx}$ is a contribution of each resonance into the dynamical partition function $\tilde{Q}$, used here as a weighting factor in the averaging. The values of $\tilde{Q}_i^{\mathrm{cov}}$, $\tilde{Q}_i^{\mathrm{vdw}}$ and $\tilde{Q}_i^{\mathrm{free}}$ are obtained by substitution of p_i^{cov}, p_i^{vdw} and p_i^{free} into this formula in place of p_i^{xxx}. Note that Γ_i is the total resonance width. The average values of resonance width in symmetric and asymmetric isotopomers were computed in the same way, but using p_i^{sym} and p_i^{asym} in place of p_i^{xxx}. Zero-pressure limit was assumed for calculations of all average values, which corresponds to $w_i = 1$ for each resonance, regardless of its width (as in $\tilde{Q}_i^{\circ}$ above).

Location of the effective transition state that separates the covalently bound ozone molecule from the weekly-bound van der Waals complex is accurately determined by inspecting the dependence of adiabatic energy (of 2D solution for hyper-angles θ and φ) as a function of hyperradius ρ. Such dependence indicates a well-defined barrier near $\rho^{\dagger} \sim 5.5$ Bohr, sensitive to the rotational excitation (quantum numbers *J* and *K*), recombination pathway under consideration, and the number of isotopic substitutions. First, the covalent well probability p_i^{cov} is obtained by integrating the vibrational wave function of scattering resonance through the range $0 < \rho < \rho^{\dagger}$. The contributions of symmetric and asymmetric ozone molecules, p_i^{sym} and p_i^{asym}, are determined using hyper-angle φ (see Fig. 4 in the main text). One third of the range in the vicinity of symmetry

axis ($2\pi/3 < \varphi < 4\pi/3$) corresponds to symmetric ozone isotopomer, while the remaining two thirds of the φ-range correspond to asymmetric ozone molecules.

A complex absorbing potential (CAP) in the form suggested by Manolopoulos [J. Chem. Phys. **117**, 9552 (2002)] was used to impose the boundary conditions. The CAP was defined with the minimum absorption energy $E_{\min} = 7$ cm^{-1} and spans the range of ~ 6 Bohr going inward from the end of the ρ-grid. The optimized DVR-grid for ρ coordinate covered the range of $3 \leq \rho \leq 15$ Bohr and consisted of 88 points. It is nearly impossible to converge every individual state above the dissociation threshold. Therefore, our convergence parameters were adjusted to ensure convergence of the overall recombination rate coefficient to within few percent. Convergence of the individual states depends on their properties. Broad resonances with $\Gamma \sim 10$ cm^{-1} are converged to within 1 cm^{-1} or better (both energy and width). Narrower resonances are converged much better. Convergence of broader resonances is not important, since they make negligibly small contributions to the dynamical partition function Q (their weights w_i are close to 1, but their probabilities p_i^{cov} are close to 0).
